# Light‐Responsive Inorganic Biomaterials for Biomedical Applications

**DOI:** 10.1002/advs.202000863

**Published:** 2020-07-17

**Authors:** Hung Pang Lee, Akhilesh K. Gaharwar

**Affiliations:** ^1^ Biomedical Engineering College of Engineering Texas A&M University College Station TX 77843 USA; ^2^ Material Science and Engineering College of Engineering Texas A&M University College Station TX 77843 USA; ^3^ Center for Remote Health Technologies and Systems Texas A&M University College Station TX 77843 USA

**Keywords:** drug delivery, light‐responsive biomaterials, photodynamic therapy (PDT), photothermal therapy (PTT), regenerative medicine

## Abstract

Light‐responsive inorganic biomaterials are an emerging class of materials used for developing noninvasive, noncontact, precise, and controllable medical devices in a wide range of biomedical applications, including photothermal therapy, photodynamic therapy, drug delivery, and regenerative medicine. Herein, a range of biomaterials is discussed, including carbon‐based nanomaterials, gold nanoparticles, graphite carbon nitride, transition metal dichalcogenides, and up‐conversion nanoparticles that are used in the design of light‐responsive medical devices. The importance of these light‐responsive biomaterials is explored to design light‐guided nanovehicle, modulate cellular behavior, as well as regulate extracellular microenvironments. Additionally, future perspectives on the clinical use of light‐responsive biomaterials are highlighted.

## Graphical abstract

A range of light‐responsive inorganic biomaterials, including carbon‐based nanomaterials, gold nanoparticles, graphite carbon nitride, transition metal dichalcogenides, and up‐conversion nanoparticles is discussed and evaluated for biomedical applications including photothermal therapy, photodynamic therapy, drug delivery, and regenerative medicine.

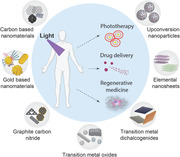

## 1 Introduction

Light has been used in modern medicine as an alternative tool for treating conditions such as skin disorders, melanoma, acne, and depression.^[^ ^1^, ^2^ ^]^ Compared to other forms of energy, such as thermal or magnetic energy, which are widely used as an external impetus to control stimuli‐responsive biomaterials, light is a particularly attractive source of energy. First, the advanced optical instruments can confine light to a pre‐defined area. Second, the degree of stimulation can be fine‐tuned by the time or intensity of light exposure. Third, light can be divided into a specific range of wavelengths, which can be independently controlled by different biomaterials to produce differential bio‐inductive cues for controlling cell–material interactions. Finally, light is a physiologically less harmful choice to control biomaterials in the physiological environment compared to temperature or pH value variation. Hence, light is a promising source of energy to control designing stimuli‐responsive biomaterials.

In the last decade, photochemistry has accelerated the development of light‐responsive biomaterials in biomedical research. Photothermal therapy (PTT) and photodynamic therapy (PDT) are emerging applications that use light in the treatment of various conditions in clinical dermatology, ophthalmology, and oncology. There are several organic photosensitizers approved by the US Food and Drug Administration (FDA) to treat certain cancers. However, one of the current challenges of using organic photosensitizers is the limited penetration depth (<1 mm) of short‐wavelength light sources used for their activation.^[^ ^3^ ^]^ Light with wavelengths in the range of 300–700 nm (UV to visible (VIS) light) is used to treat superficial tissue, and longer wavelengths in the range of 750–1100 nm (near‐infrared (NIR) light), which penetrate further, is used to treat deeper‐seated tissues.^[^ ^4^ ^]^ Therefore, to translate the research into clinical applications, the investigation of NIR‐responsive materials is more realistic for next‐generation photomedicine, which is lacking in organic photochemistry.^[^ ^5^ ^]^ Thus, NIR‐responsive inorganic nanomaterials, such as carbon‐based nanomaterials (CBNs), gold‐based nanomaterials (AuBNs), and up‐conversion nanoparticles (UCNPs), provide an useful platform to absorb and utilize NIR light for biomedical research.^[^ ^6^, ^7^, ^8^ ^]^

**Figure 1:**
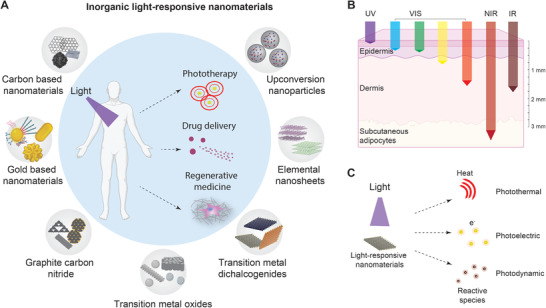
Inorganic light‐responsive nanomaterials. A) Various types of light‐responsive biomaterials include CBNs, AuBNs, g‐C_3_N_4_, TMOs, TMDs, elemental nanosheets, and UCNPs. B) The penetration depths of light into the skin depend on the wavelengths of light. UV, green, and blue light have small penetration depths less than 1 mm. In contrast, red, NIR, and IR light have longer penetration depths, and especially NIR can penetrate more than 3 mm. C) The unique optical properties of these nanomaterials convert light energy to heat, electrical stimuli, or chemical reactions, which are used for cancer therapy, drug delivery, and regenerative medicine.

In this article, we will focus on recent development (last 6 years (2014–2020)) of light‐responsive inorganic nanomaterials for biomedical applications, including PTT, PDT, drug delivery, and regenerative medicine (**Figure** **1**). We will explore the importance of these light‐responsive biomaterials to design light‐guided nanovehicle, modulate cellular behavior, as well as regulate cellular microenvironments. Apart from conventional light‐responsive biomaterials (CBNs and AuBNs), we will also discuss nanosized and exfoliated 2D nanomaterials^[^ ^9^ ^]^ such as graphite carbon nitride (g‐C_3_N_4_), transition metal dichalcogenides (TMDs), transition metal oxides (TMOs), and heterostructure nanomaterials. Additionally, we will highlight future perspectives on the clinical use of light‐responsive biomaterials.

## 2 Applications of Light‐Responsive Biomaterials

### 2.1 Photodynamic therapy (PDT) and photothermal therapy (PTT)

**Figure 2:**
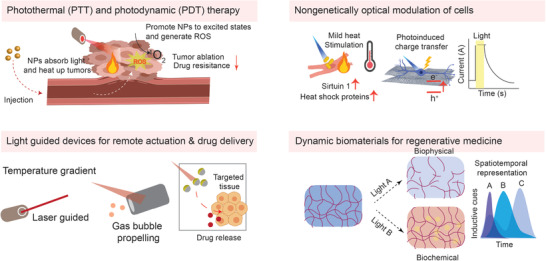
Inorganic light‐responsive nanomaterials have been widely applied to four main research fields of light‐responsive biomaterials, which consist of PTT and PDT, light‐guided devices for remote actuation and drug delivery, nongenetically optical modulation of cells, and dynamic biomaterials for regenerative medicine.

Photodynamic action is the reaction of cells to a chemical reagent, light, and oxygen. PDT is an alternative treatment for oncologic intervention through tumor cell ablation and oxidation. The first PDT research demonstrated the tremendous potential of phototherapy.^[^ ^2^ ^]^ Generally, light at a specific wavelength will excite tumor‐localizing photosensitizers to its higher excited state. A single excited photosensitizer will undergo intersystem crossing and become a triplet excited photosensitizer, which is the essential character to trigger type 1 and 2 reactions of PDT and generate free radicals and singlet oxygen, respectively (**Figure** **2**). Although the mechanisms of the two kinds of reactions are slightly different, their oxidized products have almost similar cytotoxicity toward tumor cells. In comparison to conventional chemotherapy, PDT exhibits significant advantages in that the light‐responsive materials are nontoxic and can be activated spatiotemporally, unlike chemotherapy drugs which might cause systemic toxicity to the surrounding healthy cell. Also, photosensitizers are reusable; therefore, they can improve patients’ quality of life with minimal invasiveness. There are several photosensitizers approved for PDT treatment by the FDA for various types of cancers.^[^ ^10^ ^]^ Among these photosensitizers, porfimer sodium (Photofrin) is widely used for treating patients with Barrett's esophagus, esophageal cancer, and endobronchial cancer.^[^ ^10^ ^]^ However, the wavelengths of light for activating most of the photosensitizers used in clinical are in the UV or visible light range. Therefore, NIR‐activated PDT can widen the scope of clinical applications for PDT.

Recently, many direct NIR‐responsive photosensitizers and indirect NIR‐responsive composites have been developed.^[^ ^11^ ^]^ The indirect photosensitizers are composed of a UV‐ or visible light‐responsive photosensitizers and an up‐conversion nanomaterial. The direct NIR‐responsive photosensitizers include NIR‐responsive organic and inorganic photosensitizers, which can directly transform light energy into the production of radical agents.^[^ ^12^ ^]^ However, most NIR‐responsive organic molecules and dyes have relatively low extinction coefficients and poor photostability, which can hinder their clinical applications.^[^ ^13^ ^]^ On the contrary, NIR‐responsive inorganic photosensitizers have less photobleaching effects and can be easily functionalized, with additive benefits for targeting tumor cells.

Similar to PDT functioning, PTT is considered an alternative medicine for oncology through the thermal ablation of tumor cells. Although hyperthermia treatment has already been used for treating musculoskeletal symptoms since the 19th century, the concept of PTT was not formulated until the mid‐1990s. PTT can be classified into three categories: irreversible injury treatment, hyperthermia treatment, and diathermia treatment, which is based on the magnitude of the temperature enhancement and the duration of the treatment.^[^ ^14^ ^]^ Among them, hyperthermia treatment is the most frequently used PTT for cancer therapy. A PTT treatment with temperature range of 41 and 48 °C leads to aggregation and denaturation of proteins in cancer cells, which increases the susceptibility of tumor cells to chemotherapy and irradiation. Therefore, PTT can be used as a supporting treatment in the clinic by increasing the efficacy of chemotherapy while reducing its side effects. Also, with the advancement of nanotechnology, the role of photothermal agents in cancer therapy has become more important, owing to the improved heat conversion efficacy and localized heating performance of photothermal nanoparticles. Compared to inorganic nanomaterials, most organic photothermal molecules have lower energy conversion, poor photothermal stability, and complicated synthesis processes. Thus, NIR‐responsive inorganic nanomaterials have become mainstream in the development of PTT in recent years.

Inorganic nanomaterials have been incorporated to improve PTT even further.^[^ ^15^ ^]^ For example, the PTT based on gold nanoparticles (AuNPs) has recently shown promise for treating prostate cancer.^[^ ^16^ ^]^ Subsequently, the PTT combined with surgery and radiation as a novel synergistic therapy showed enhanced treatment efficacy.^[^ ^16^ ^]^ Furthermore, inorganic nanomaterials could be covalently or noncovalently modified by functional biomolecules due to their large surface‐to‐volume ratio. For instance, the addition of targeting ligands to the surface of photothermal nanomaterials can improve their permeability and retention in tumors, and therefore minimize the side effects of heat treatment on the surrounding healthy tissue. The enhanced target specificity of PTT agents could also help inactivation of the unhealthy cells infected by viruses and bacteria to accelerate patient recovery.^[^ ^17^ ^]^ Even though gold and carbon‐based materials have been intensively investigated in the field of PTT, they still have the main drawback of showing a weak fluorescence, which limits their application in visual‐guided therapies.^[^ ^18^ ^]^ Several emerging nanomaterials, such as TMDs and elemental nanosheets, have been investigated to improve PTT performance even further, to treat tumors without surgical intervention eventually.^[^ ^18^ ^]^

### 2.2 Nongenetic Optical Modulation of Cells

Light‐responsive nanomaterials can be used for cell modulation, especially for cardiac cells and neurons. Using sophisticated light‐controlled platforms, a single cell can be modulated by inorganic nanomaterials through photothermal or photoelectric stimulation.^[^ ^19^, ^20^ ^]^ This type of single‐cell approaches allows detection or on‐demand trigger of cellular processes.^[^ ^21^ ^]^ Extracellular stimulation of excitable cell types such as neurons, cardiomyocytes, and skeletal muscle cells, can treat several types of diseases.^[^ ^20^ ^]^ For instance, photovoltaic retinal prosthetics could ameliorate the symptoms of retinitis, which are caused by losing the ability to transduce light into electrical signals, as the replacement of light‐sensing tissue.^[^ ^22^ ^]^ Functionalized inorganic nanomaterials can be easily delivered and dispersed in a drug‐like manner to targeted locations in deep brain or heart tissue. Also, NIR light can remotely control these photoactive materials to transduce energy to excite cells in deep tissue layers. Moreover, the optical cell modulation by the single‐cell assay also can provide cellular or intercellular‐scale measurements to understand the functional diversity of the cells and identify phenotypically rare cells, rather than the overall result of treatment.^[^ ^23^ ^]^

There are two main types of signals that were used to control cell behavior: electrochemical and temperature. The photothermal effects of inorganic nanomaterials and the ability to apply thermal stimulation on a single cell have been well established to understand the effect of small change of temperature influenced cellular function.^[^ ^24^ ^]^ For example, the thermal stimulation caused by external laser irradiation can activate or inhibit temperature‐sensitive gene expression, neuron activity, and cardiomyocyte beating.^[^ ^20^, ^25^ ^]^ However, it is still unknown if long‐term chronic thermal exposure will cause side effects in tissues. Thus, photoelectrochemical or photovoltaic stimulation could be an alternative way to study the nongenetic modulation of cell behavior, which is less damaging and more sensitive to cells.

In recent years, many efforts have been made to scale down photovoltaic materials into freestanding nanoscale devices. Quantum dots (QDs) and semiconductor nanomaterials have been applied to stimulate electrical triggerable cells.^[^ ^26^ ^]^ These light‐responsive nanomaterials could provide a platform to study how thermal or electrical stimulation controls the subcellular activity of gene transcription and protein signaling pathways.^[^ ^27^ ^]^ Moreover, external electrical stimulation, delivered by traditional bioelectrodes, has been used in treating several clinical symptoms of electrophysiological nature, such as cardiac arrhythmias and Parkinson's disease.^[^ ^28^ ^]^ Therefore, through translating the results of these fundamental research to clinical treatments, light‐responsive nanomaterials can provide a less‐invasive and more precise strategy to treat these electrophysiological disorders.

### 2.3 Light‐Guided Devices for Remote Actuation and Drug Delivery

Micro/nanomotors are miniaturized devices that allow precise manipulation of materials on a cellular scale.^[^ ^29^ ^]^ Recently, light‐guided nanovehicle is a unique type of micro/nanomotors, which have applied to develop active on‐demand delivery systems to transport cells, drugs, and biomolecules to the targeted tissue. Due to their nanoscale sizes, nanovehicle can penetrate cell membranes to accomplish intracellular transport. However, their biocompatibility is the most challenging issue, as most nanovehicles rely on asymmetric surface chemical reactions to generate ions and electric fields to obtain a propulsion force.^[^ ^30^ ^]^ This limits the selection of supporting chemicals. Hydrogen peroxide is one of the most commonly used supporting chemicals to propel nanovehicles; however, it is often not a practical chemical choice for biomedical uses. Moreover, due to the high ionic strength, high viscosity, and plasma protein, the physiological environment is even more challenging for propelling nanovehicles. Alternative strategies utilize external stimuli, such as magnetic fields, electrical fields, and ultrasound, to propel nanovehicles.^[^ ^31^ ^]^

Light‐guided nanovehicle has become a promising solution to accomplish active delivery in the physiological microenvironment. Additionally, in such scenarios, light can provide additional degrees of freedom to manipulate light‐responsive materials by controlling light intensity, frequency, polarization, and direction with high spatial and temporal precision.^[^ ^32^ ^]^ Thus, light‐responsive materials, such as photoelectrochemical cells or heating agents, can absorb photon energy in a fuel‐free physiological environment to generate an electrochemical or temperature gradient. For instance, asymmetric photocatalytic heterostructures, including Au/TiO_2_, Au/Fe_2_O_3_, and Au/Cu_2_O nanomaterials_,_ can generate self‐electroosmosis flow via the separation of electron–hole pairs in a low concentration of hydroxy peroxide and by a low power density of UV irradiation.^[^ ^33^ ^]^ The existing knowledge of photocatalytic heterostructure nanomaterials can also readily apply to develop light‐guided nanovehicle, as the heterostructures have already been used to increase the efficiency of photocatalysts.

In addition to photoelectrochemical nanomaterials, photothermal nanomaterials also have attracted interest in the development of light‐guided nanovehicles. The photothermal effect of light‐guided nanovehicles can generate a local temperature gradient to propel Janus microcapsule motors, whose surface should have two or more distinct physical properties. Gold is one of the most popular materials, and it can be asymmetrically coated or sputtered on any nanoparticles, such as magnetic Fe_2_O_3_, mesoporous silica, or TiO_2_. These synthesized heterojunction nanomaterials are multifunctional, which can be used in light‐guided chemo‐photothermal therapy as well. Moreover, photothermal nanomaterials can also be integrated with thermal‐responsive molecules or polymers, such as poly(*N*‐isopropyl acrylamide), to process body deformation to move and function as soft robots.^[^ ^34^ ^]^ Overall, guided‐devices is a novel technique combining the knowledge of PDT, PTT, nanofabrication, and nanoarchitecture, and holds great potential for precise microsurgery and cellular drug delivery.

### 2.4 Dynamic Biomaterials for Cellular Biology and Regenerative Medicine

Dynamic biomaterials add spatiotemporal tunability to a 3D biomaterial matrix by providing dynamic extracellular biophysical and biochemical cues to influence cellular behavior. A range of such light‐responsive biomaterials is currently evaluated to understand cellular biology and tissue engineering.^[^ ^3^ ^]^ For example, the changes in stiffness of light‐responsive hydrogel can influence cancer cell migration dynamically.^[^ ^35^ ^]^ Mechanotransduction is a process through which extracellular matrix (ECM) sends signals to cells to remodel the microenvironment mechanically.^[^ ^36^ ^]^ The stiffness of ECM is known to influence stem cell phenotype and cellular invasion.^[^ ^35^ ^]^ Due to the broad range of the time scales needed for the alteration in the ECM stiffness, the dynamic alteration of stiffness by an external source is necessary. The secretion and transportation of biomolecules also induce the heterogenous structure and functions of the natural cellular microenvironment. Another study reported a platform to control the adhesion of cells by light‐triggered activation of a cell‐adhesive RGD peptide, thus demonstrating the possibility of modulating aspects of tissue regeneration of implanted biomaterial in a spatiotemporal manner.^[^ ^37^ ^]^ Growth factors delivered and released by light‐responsive nanoparticles in a hydrogel matrix may promote cell proliferation on demand. Therefore, the recent progress in developing novel applications for light‐responsive biomaterials in 3D cell culture holds great promise in future translational clinical applications.

## 3 Inorganic Light‐Responsive Biomaterials

### 3.1 Carbon‐Based Nanomaterials

**Figure 3:**
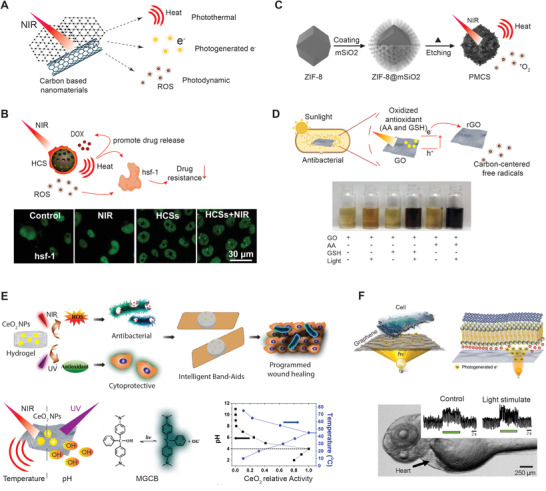
CBNs are widely used as light‐responsive biomaterials. A) CBNs can generate heat, electrons, and ROS for various biomedical applications. B) HCS is used as a photodynamic agent and drug carrier to overcome chemotherapy resistance by suppressing drug resistance‐related genes via the production of hsf‐1 protein homotrimers. The fluorescent microscopy images show the change in the number of hsf‐1 protein homotrimers in the nucleus after NIR laser irradiation. Adapted with permission.^[^ ^44^ ^]^ Copyright 2015, American Chemical Society. C) Mesoporous carbon nanosphere contains porphyrin‐like zinc centers obtained from an imidazolate framework (ZIF‐8) precursor by a surface‐protected pyrolysis strategy. Adapted with permission.^[^ ^45^ ^]^ Copyright 2016, John Wiley & Sons. D) Simulated sunlight accelerates transferring electrons to GO from the antioxidant biomolecules that protect bacteria from ROS. Photographs of GO dispersions under various conditions: sunlight alone, antioxidant (glutathione (GSH) and ascorbic acid (AA)) alone, or antioxidant combined with sunlight. The transferred electrons can reduce GO only in the presence of antioxidants and sunlight simultaneously. Adapted with permission.^[^ ^47^ ^]^ Copyright 2017, American Chemical Society. E) CeO_2_ nanoparticle‐embedded hydrogels can accelerate the multi‐phases of the wound healing process by the precise control of chemical reactions. The photothermal effect of graphene oxide and the pH regulation of malachite green carbinol base (MGCB) can control CeO_2_ nanoparticles’ catalytic activity of being an oxidant or antioxidant for wound healing. NIR light promotes the production of ROS to kill bacteria, and UV light increases the production of antioxidants to protect cells against oxidative stress. Adapted with permission.^[^ ^48^ ^]^ Copyright 2017, American Chemical Society. F) Graphene is used as an optoelectronic interface for optical stimulation of cells. The photogenerated electrons control the heart rates of zebrafish embryos by optical stimulation using a wide range of light wavelengths. Adapted with permission.^[^ ^52^ ^]^ Copyright 2018, American Association on for the Advancement of Science (AAAS).

CBNs have been widely investigated and applied in almost every area of engineering research. Since fullerene was first discovered in 1985, various shapes of CBNs have been revealed, such as graphene, carbon nanotubes (CNTs), graphene oxide (GO), nanodiamonds, and carbon QDs.^[^ ^38^ ^]^ Due to their superior electrical, magnetic, optical, mechanical, chemical, and biocompatible properties, CBNs play a significant role in nanobiotechnology from cancer therapy to regenerative medicine. In the 1990s, the carboxylic acid‐functionalized fullerene was the first synthesized CBNs and has shown its potential ability for PDT.^[^ ^39^ ^]^ The surface‐functionalized CBNs are water‐soluble, biocompatible, and multifunctional and can function as a PDT, PTT, and drug carrier agent simultaneously (**Figure** **3**A).

CNTs are 1D pipe‐type fullerene and can be classified based on the number of graphite layers into single‐walled nanotubes (SWNTs) and multi‐walled nanotubes (MWNTs). MWNTs absorb light approximately four times greater than SWNTs resulting in higher photothermal conversion efficiency for MWNTs.^[^ ^40^ ^]^ On the other hand, semiconducting SWNTs could generate radical oxygen species (ROS) more than metallic SWNTs due to the suitable bandgap energy of semiconducting SWNTs, which shows great potential for PDT.^[^ ^41^ ^]^ Moreover, CNTs can conjugate biomolecules and have a massive drug‐loading capacity of their hollow structure via *π*–*π* stacking as multifunctional carriers for cancer therapy.

Graphene is a 2D material featuring sp^2^ hybridization with honeycomb hexagonal structure, which has a stronger photothermal activity compared to CNTs. Other than photothermal cancer therapy, graphene‐based photothermal antiviral therapy was recently reported.^[^ ^17^ ^]^ Specific virion glycoproteins of Herpes Simplex Virus can bind heparin sulfate moieties on the surface of the host cells, which help promote virion attachment and entry into susceptible cells.^[^ ^17^ ^]^ Sulfonate‐functionalized graphene can mimic the heparin sulfate moieties to capture viruses and then inactivate them by PTT.^[^ ^17^ ^]^ Although photothermal antiviral therapy still is at an early development stage, PTT can be an alternative and efficient method to combat viral infections with proper surface functionalization.

Graphene possesses excellent light‐to‐heat conversion ability, but its relatively low solubility could be a hindrance to its practical use in clinical. Therefore, GO would be a better option of 2D CBNs because it has better biocompatibility and higher solubility in water through facile surface functionalization, which has been extensively exploited for phototherapy. Combining its photothermal effect and the *π*–*π* interaction with a drug, GO was regarded as a promising nanomaterial for an NIR drug‐releasing system. On the other hand, the *π*–*π* interaction might limit its drug‐loading capacity because it is highly dependent on the chemical structure of the drug. Thus, in recent years, thermal responsive polymers and silica nanoparticles have been integrated with GO to enhance their drug‐loading capacity and spatiotemporal precision of drug delivery.^[^ ^42^ ^]^ A light‐responsive drug release system was developed via coating the mixture of fatty alcohol, a phase change material, and doxorubicin on the graphene oxide and silica nanocomposite.^[^ ^43^ ^]^ The melting point for a phase change material is around 38 °C, which allows rapid drug‐release by NIR irradiation, and the hydrophobicity of graphene and the fatty alcohol nanocomposites improves the performance of the nanocarrier internalization.^[^ ^43^ ^]^ This research presented a novel light‐responsive drug‐delivery system without complicated synthesis conditions and toxic side products, thus proving to be an ideal material for clinical use.

Recently, hollow carbon nanospheres (HCSs), with high load capacity, can deliver a large number of drug molecules and perform PTT and also generate ROS to disrupt the cellular redox state (Figure 3B).^[^ ^44^ ^]^ The HCSs also feature sp^2^ and sp^3^ bonded carbon atoms equipped with excellent catalytic activity to generate free radicals under NIR light irradiation, which can activate heat shock factor‐1 (hsf‐1) gene expression to overcome chemotherapy resistance.^[^ ^44^ ^]^ For combinational phototherapy of PDT and PTT, a metal‐organic framework (MOF) was incorporated into mesoporous carbon nanospheres.^[^ ^45^ ^]^ The MOFs were synthesized by carbonization of a zeolitic imidazolate framework (ZIF‐8), where mesoporous‐silica was used to prevent the aggregation of nanospheres during the high‐temperature pyrolysis. The zinc and nitrogen co‐doped carbons (Zn‐N‐C) could be used as stable photosensitizers to generate ^1^O_2_ via electronic transfer from the conjugated *π*‐bond of a porphyrin‐like structure to molecular oxygen, yielding a similar quantity of singlet oxygen quantum to ICG (a common NIR dye) (Figure 3C). Due to its strong NIR absorption, it also had excellent IR and photoacoustic (PA) imaging contrast, allowing real‐time monitoring of the therapeutic process, thus establishing it as a potential material for conformal cancer therapy.

Besides cancer therapy, CBNs are also known for their antibacterial function, in which GO showed the highest antibacterial activity of generating cell membrane stress among graphite, GO, and reduced GO.^[^ ^46^ ^]^ Moreover, it has been reported that under simulated sunlight, GO's antibacterial activity was enhanced by the accelerated electron transfer from the innate antioxidant systems of *E. coli* to GO, but not via the mechanisms of PDT or PTT.^[^ ^47^ ^]^ The only type of ROS induced by light exposure was singlet oxygen (^1^O_2_), which had a slight impact on the oxidation of antioxidants. Surprisingly, the light was able to generate electron–hole pairs by transferring electrons from antioxidants (glutathione and ascorbic acid) to reduced GO, along with introducing carbon‐centered free radicals to kill bacteria (Figure 3D). To date, the interaction between light and CBNs and its effects in a biological environment, are not fully understood. Thus, to further elucidate the roles of CBNs as phototherapy agents, their light‐induced activities need to be investigated in more detail.

Temperature is known as a critical parameter to control chemical reactions in order for photothermal nanomaterials, such as graphene, to precisely control reaction activity. A dual light‐responsive system was developed to fine‐tune temperature and pH, in order to regulate the chemical reaction of a hydrogel to promote wound healing (Figure 3E).^[^ ^48^ ^]^ In this research, GO was integrated with malachite green carbinol base (MGCB), which can produce hydroxide and increase pH value under UV irradiation. In the absence of UV light, and only NIR light irradiation present, GO‐MGCB‐agarose hydrogel exhibited the highest antibacterial activity because the acidic environment promoted the activity of CeO_2_ nanoparticles as oxidants. The photothermal effect of GO also contributed to this antibacterial effect. In contrast, to promote cell proliferation, the high pH value (due to MGCB) enabled CeO_2_ nanoparticles to exhibit high antioxidative activity under UV irradiation. This research is a proof‐of‐concept for introducing inorganic nanomaterials to construct a multi‐wavelength light‐responsive and multifunctional biomaterial. Besides chemical reactions, the incorporation of thermal responsive polymers or thermotropic liquid‐crystalline elastomer (LCE) can improve the photothermal effect of CBNs to help control the shape of the materials in order to achieve potential applications such as 4D bioprinting, artificial muscle, and smart actuators.^[^ ^49^, ^50^, ^51^ ^]^ In order to perform these applications, photoresponsive hydrogels and polymers should have appropriate mechanical properties, such as high tensile strength and toughness. For example, GO could also be considered as a physical crosslinker to avoid the use of small molecule crosslinkers, and to prevent the formation of highly crosslinked dense clusters, which can cause inhomogenous deformation. Instead, the hydroxyl and carboxyl groups of GO could form hydrogen bonds to dissipate the strain energy when the hydrogel is under deformation.^[^ ^50^ ^]^ GO‐doped LCE also demonstrates the importance of the distribution and interaction of 2D nanomaterials within polymers to transfer load efficiency and shorten the responsive time of actuating.^[^ ^49^ ^]^ The aromatic rings and ester groups of LCE can assist GO to have a uniform dispersion via *π*–*π* stacking and hydroxyl bond formation. Moreover, the combination of in situ polymerizations of liquid‐crystalline monomers and concurrent hot‐drawing processes results in the formation of highly aligned GO sheets. Additionally, the solvothermal‐restored GO has a higher degree of sp^2^‐bonded carbon with higher thermal conductivity and more efficient heat transport via GO lattice vibration, thus resulting in considerable higher contraction strain, high actuation stress, and fast response of GO/LCE composites.^[^ ^49^ ^]^ However, all these examples of photothermally induced actuators carry similar concerns regarding heat damage and poor biocompatibility for in vivo use.

Graphene is not only a photothermal agent but also a nanophotovoltaic device. Graphene has zero bandgap and strong electron–electron interaction making it a highly efficient light‐to‐electricity converter to modulate cell behavior optically.^[^ ^52^ ^]^ The first study of optical stimulation of cardiomyocytes in a substrate‐based and dispersible configuration were reported via a graphene bio‐interface. Light illumination could trigger cell membrane depolarization on the graphene‐coated substrate, which was confirmed by the electrophysiological observation of action potential generation and the increase in action potential frequency (Figure 3F). Moreover, graphene could eject the photogenerated electrons with a mean free path of up to 1 µm allowing it to overcome the limited mechanical conformity of graphene due to the poor match between the curvature of a flake and the shape of a cell. In addition, the generation of hot‐carriers could be detected in any light wavelength from 300 to 2500 nm for optical stimulation. Optical stimulation of the zebrafish heart accompanied by the injection of graphene solution resulted in a significant increase in heart rate.

### 3.2 Gold‐based Nanomaterials (AuBNs)

AuBNs have been used in biomedical engineering for a long time due to their significantly lower toxicity, high conductivity, chemical stability, and feasibility of the synthesis, deformation, and functionalization.^[^ ^53^ ^]^ It is also well‐known for its optical property of localized surface plasmon resonance (LSPR), which is a collective oscillation of conduction band electrons in metal nanoparticles excited by the electromagnetism of the incident light. Mechanistically, the resonance can enhance light absorption and cause strong local electromagnetic fields on the AuBN surface, among other optical phenomena. The LSPR bands can be extended to the NIR range by tuning the shape and aspect ratio of AuBNs because of the surface photon confinement effect. Therefore, AuBNs with the LSPR peaks in the NIR range have been thoroughly investigated for the last two decades for biomedical applications such as PTT and PDT.

**Figure 4:**
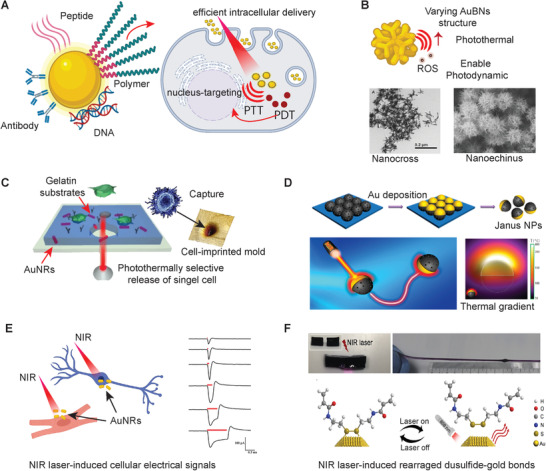
AuBNs are well known for their optical property of localized surface plasmon resonance used to develop various light‐responsive biomaterials. A) The surface modification of AuBNs brings efficient intracellular delivery for photothermal and PDT, which the heat and ROS generated by AuBNs directly attack the nucleus. B) Versatile structures of AuBNs, such as nanocross and nanoechinus, equip with different optical properties, which enhance the photothermal effect and enable photodynamic reaction. Adapted with permission.^[^ ^60^ ^]^ Copyright 2016, John Wiley & Sons. Adapted with permission.^[^ ^61^ ^]^ Copyright 2014, John Wiley & Sons. C) Cell is captured and released by AuBNs embedded gelatin substrate for single‐cell assay. Adapted with permission.^[^ ^65^ ^]^ Copyright 2016, American Chemical Society. D) Janus gold‐sputtered nanoparticles use the gradient of heat produced by the photothermal effect to move. Adapted with permission.^[^ ^66^ ^]^ Copyright 2016, American Chemical Society. E) AuNPs use mild heat pulses to simulate neuron and cardiomyocytes by the photothermal effect. Adapted with permission.^[^ ^67^ ^]^ Copyright 2014, John Wiley & Sons. F) NIR laser can rearrange the bonds between the disulfide groups of polymers and the surface of AuNPs. Optical images are showing the self‐healing procedure between two separated gold nanocomposite hydrogel pieces under an NIR laser. Adapted with permission.^[^ ^69^ ^]^ Copyright 2017, Elsevier.

Among AuBNs, gold nanorods (AuNRs) are considered as one of the most promising agents for PTT and tumor diagnosis, owing to their tunable and strong NIR absorption. By changing the concentrations of silver ions, the aspect ratio of AuNRs can be controlled, which results in different band positions and colors of solutions.^[^ ^54^ ^]^ The efficiency of PTT is limited by the maximal permissible exposure (e.g., 0.33 W cm^−2^ for 808 nm) in human skin by the American National Standards Institute, due to the potential danger of the skin burning and damage to adjacent healthy tissue.^[^ ^55^ ^]^ Therefore, there is a high demand for extremely low NIR light intensity‐activated PTT. Various surface modifications, such as dispersant, peptide, DNA, and antibody, can enhance the targeting efficacy of AuBNs to decrease the required intensity of NIR light (**Figure** **4**A). TAT peptides carrying nuclear localization signals were conjugated onto AuNRs surfaces for intranuclear delivery, which could directly break DNA to suppress damages to surrounding tissue.^[^ ^56^ ^]^ The same strategy could also be applied to NIR‐activated PDT demonstrated by nucleus‐targeting gold nanoclusters, which could sensitize the formation of singlet oxygen upon photoexcitation of short NIR light (808 nm).^[^ ^57^ ^]^ The generated ROS has very high chemical reactivity with a limited lifetime and diffusion range, which leads to the direct targeting of the cell nucleus and inducing DNA damage, making it one of the most effective forms of PDT (Figure 4A).^[^ ^57^ ^]^ Besides cancer therapy, PDT and PTT are alternative treatments for Alzheimer's disease.^[^ ^58^ ^]^ The synthesized penetratin peptide allowed gold nanostars to penetrate the blood–brain barrier to perform PTT of Alzheimer's disease, demonstrating the inhibition and dissociation of amyloid‐beta fibrils formation.^[^ ^58^ ^]^ The penetratin‐conjugated gold stars demonstrated excellent neuroprotective effects against cellular toxicity under NIR irradiation.^[^ ^58^ ^]^

The anisotropic AuBNs with multiple branches have better photothermal efficiency than the spherical or multifaceted AuBNs because of the synergistic enhancement of LSPR (Figure 4B).^[^ ^59^ ^]^ Gold nanocrosses modified with antibodies for targeting proteins on multidrug‐resistant bacteria could also be used to ablate these bacteria due to the enhancement of photothermal efficacy.^[^ ^60^ ^]^ Nanomaterials able to perform PDT under NIR irradiation are rare, especially in both biological windows I (650–950 nm) and II (1000–1350 nm). Most PDT agents activated in the NIR range depend on the combination of up‐conversion agents and organic photosensitizers, which might have problems of photobleaching and low efficacy. Another study designed a multi‐branched gold nanochinus, which could perform both PDT and PTT in the second biological window to destruct deep‐tissue tumors by the generation of singlet oxygen (Figure 4B).^[^ ^61^ ^]^

AuBNs are one of the most well‐known nanomaterials for phototherapy that have been investigated for a wide range of applications. In recent years, scientists hoped to use this stable, bioinert, and easily synthesized nanomaterials to solve complicated biomedical problems by functionalizing surface properties, designing new shapes, and synthesizing nanocomposites of AuBNs. Usually, AuNPs are synthesized using reducing agents by the reduction of HAuCl_4_. The hydroxyproline residues of collagen chains can also be used to reduce AuCl_4_ ions, which could simultaneously trigger the self‐assembly of collagen fibers via electrostatic interaction.^[^ ^62^ ^]^ Also, the collagen‐gold hydrogel displayed shear‐thinning and self‐healing properties via weak reversibly electrostatic interactions, which were used to develop the injectable hydrogel for PTT and PDT.^[^ ^62^ ^]^

Composites of AuBNs and thermal‐responsive polymers are accessible materials to fabricate light‐controlled actuators and artificial muscle.^[^ ^63^, ^64^ ^]^ Doping AuNRs into UV‐visible responsive azobenzene liquid‐crystalline showed the proof‐of‐concept of a light‐responsive soft robot by demonstrating the biomimetic motion of push‐ups and sit‐ups.^[^ ^63^ ^]^ The photomechanical effects of the soft robot were achieved by two types of photoresponsive molecular motion—photothermally induced phase transition and trans‐cis photoisomerization of azobenzene mesogens. Moreover, light‐controlled phase transitions of gelatin by incorporation of photothermal AuNRs could apply to isolate and capture single circulating tumor cells for promoting the process of individualized antitumor therapies (Figure 4C).^[^ ^65^ ^]^ The imprinted cell‐shape microstructure and antibody‐coated substrate could help capture the targeted cancer cells. Then, an NIR laser could trigger the photothermal effect of AuNRs to process the sol–gel transition of gelatin, which could selectively release the cells for gene mutation analysis.^[^ ^65^ ^]^ This study showed that the mild heat generated by the photothermal effect of AuBNs is not only limited to cancer therapy but also has the potential to perform cell delivery for tissue regeneration.

Inspired by the self‐thermophoresis mechanism, gold was sputtered on the half‐shells of mesoporous silica nanoparticles, which could generate a thermal gradient via NIR irradiation without adding fuel to propel nanoparticles forward (Figure 4D).^[^ ^66^ ^]^ Therefore, the direction and speed of nanoparticles could be controlled by NIR intensity. The high drug‐loading capacity and biocompatibility of mesoporous silica make it a suitable candidate as a next‐generation nanovehicle for drug delivery.^[^ ^66^ ^]^ However, due to the high resistance of blood flow, the application of nanovesicles in vivo requires high propulsion force, the generation of which requires high NIR intensity. This high NIR intensity can lead to excess heat generation, which might cause damage to the skin, blood vessels, and surrounding tissue.

Transient heating generated by photothermal nanomaterials within a confined area can also stimulate the electrical activities of excitable cells. Conventionally, infrared neural stimulation (INS) uses transient (in the order of a millisecond) and localized heating, generated by the NIR absorption of water, to stimulate neurons. Therefore, AuBNs are promising candidates of INS due to their relatively small size and superior biocompatibility, which allows them to bind to the cell membrane to perform precise single‐cell stimulation (Figure 4E).^[^ ^67^ ^]^ Silica‐coated AuNRs cultured with primary auditory neurons could absorb NIR laser (780 nm) to stimulate the neurons by heating.^[^ ^67^ ^]^ Moreover, the observed electrical activity of the stimulated neurons correlated with the temperature variations indicating that the PTT might have the potential to be used as a therapeutic method for neuron regeneration.^[^ ^67^ ^]^ The capability of remotely controlling the myotube contraction of muscle cells induced via the photothermal effect of AuNRs was demonstrated.^[^ ^25^ ^]^ The results showed that the photothermal stimulation significantly enhanced the mRNA of genes encoding heat shock proteins and Sirtuin 1, a protein that can induce mitochondrial biogenesis.^[^ ^25^ ^]^ Besides neural and muscle tissues, AuNPs can also control the beating of cardiomyocytes. NIR absorption by AuNPs can generate heat pulses causing the contractions of skinned cardiomyocytes in free Ca^2+^ solution.^[^ ^68^ ^]^ Therefore, mild heat stimulation by AuNRs has provided an innovative platform in regenerative medicine for excitable tissues.

The combined treatment of PTT and light‐triggered drug delivery have shown the enhanced efficiency of antitumor chemotherapeutics. Through examining and minimizing the damages caused by PTT, the technique of photothermally localized drug delivery can also be applied to the biochemical alteration of 4D cell culture. Moreover, to dynamically control mechanical properties, another study developed a self‐healing nanocomposite hydrogel using gold nanoparticles as multifunctional crosslinkers, where the disulfide contained polymers conjugated with AuNPs to form a 3D network (Figure 4F).^[^ ^69^ ^]^ Once the hydrogel was exposed to an NIR laser, the disulfide‐gold bonds would rearrange and promote a self‐healing process.^[^ ^69^ ^]^ Meanwhile, the mechanical properties of the hydrogels could be tuned by the degree of crosslinking and laser exposure.^[^ ^69^ ^]^ Although AuBNs have been discovered over three decades ago, the inertness, light‐responsiveness, and biocompatibility of AuBNs make them still attractive in light‐controlled cancer therapy and tissue engineering.

### 3.3 Graphite carbon nitride (g‐C_3_N_4_)

**Figure 5:**
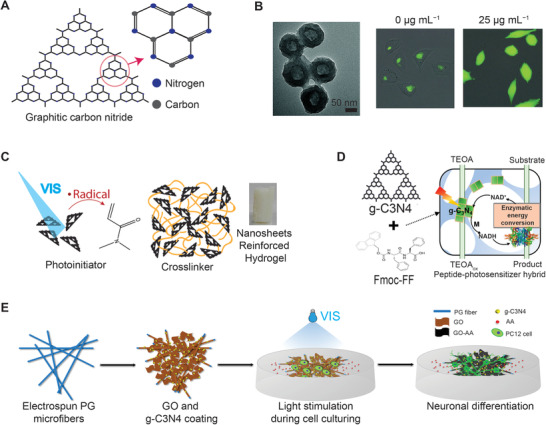
g‐C_3_N_4_ is a visible light‐responsive photosensitizer. A) g‐C_3_N_4_ has a high‐degree condensation of the tri‐s‐triazine ring structure. B) TEM images of g‐C3N4 nanospheres that can be used as a photodynamic agent and drug carriers for the synergistic therapy of PDT and chemotherapy. The fluorescence microscopy images show the light‐induced ROS production in the cells treated with 0 and 25 µg mL^−1 ^g‐C_3_N_4_ nanospheres. Adapted with permission.^[^ ^72^ ^]^ Copyright 2015, Royal Society of Chemistry. C) g‐C_3_N_4_ nanosheets are used as photoinitiators and crosslinkers to form hydrogels reinforced by the nanosheet structure of g‐C_3_N_4_. Adapted with permission.^[^ ^75^ ^]^ Copyright 2017, American Chemical Society. D) Self‐assembled peptide (Fmoc‐FF)/ g‐C_3_N_4_ hydrogels can perform biomimetic photosynthesis to reduce NAD^+^ to enzymatically active NADH and drive light‐responsive redox biocatalysis. Adapted with permission.^[^ ^76^ ^]^ Copyright 2017, American Chemical Society. E) g‐C_3_N_4_ can stimulate neuronal differentiation via light stimulation with the help of graphene oxide (GO). Adapted with permission.^[^ ^77^ ^]^ Copyright 2017, American Chemical Society.

g‐C_3_N_4_ is a visible light‐responsive nanomaterial which is composed of tri‐s‐triazine mostly made by the polymerization of cyanamide, dicyandiamide, melamine, urea, or thiourea under different reaction conditions and temperatures (**Figure** **5**A).^[^ ^70^ ^]^ g‐C_3_N_4_ has an appealing electronic structure with a medium bandgap (2.7 eV), which has been used in various applications ranging from photocatalytic to photoelectronic materials. Moreover, g‐C_3_N_4_ has been synthesized in different shapes, including nanosheets, hollow structure, and mesoporous structure, to increase its photocatalytic activity.^[^ ^71^ ^]^ For instance, the hollow g‐C_3_N_4_ sphere enhanced ROS generation for PDT, and the uniform hollow structure allowed for the storage of drugs as a novel photochemical internalization strategy (Figure 5B).^[^ ^72^ ^]^ The fluorescence microscopy images showed light‐induced ROS production of the breast cancer cells treated with 0 and 25 µg mL^−1^ hyaluronic acid‐coated hollow g‐C_3_N_4_ nanosphere under visible illumination.^[^ ^72^ ^]^ Photochemical internalization is a new method to overcome endo/lysosomal sequestration via co‐localizing drugs and photosensitizers, which generates ROS to cause lipid peroxidation and active release of drugs into the cytoplasm.^[^ ^72^ ^]^

The photocatalytic activity of g‐C_3_N_4_ could also be enhanced by embedded noble metal nanoparticles that act as electron acceptors to inhibit the fast recombination of the photoexcited electron–hole pairs. The Ag nanoparticles‐doped g‐C_3_N_4_ could have led to higher production of ROS, which enhanced its antibacterial and biofilm‐disrupting ability under visible light irradiation.^[^ ^73^ ^]^ Photopolymerization is an essential process for the curing of a dental filling, 3D bioprinting hydrogel, and forthcoming 4D bioprinting. Therefore, in the past decade, new photoinitiators activated at the near UV and visible ranges drew much attention due to less cell and material damage during photolysis. Due to the large surface area of the mesoporous g‐C_3_N_4_, the enhanced photoactivity also allowed g‐C_3_N_4_ to perform photopolymerization as a stable and cheap photoinitiator via visible light illumination from two 50 W white light‐emitting diode.^[^ ^74^ ^]^ Various types of g‐C_3_N_4_ synthesized from different precursors were compared to obtain different surface area, surface zeta potential, and C:N ratios of g‐C_3_N_4_ nanosheets, with these properties having a strong influence on the gelation time and mechanical strength of the photopolymerized nanocomposite hydrogels.^[^ ^75^ ^]^ g‐C_3_N_4_ could directly participate in the gelation process as an initiator, solid reinforcer, and colloidal cross‐linker by producing radicals on its surface where they could also act as anchoring points for the polymer chain (Figure 5C).^[^ ^75^ ^]^ Moreover, the negatively charged surface and 2D nanostructure of g‐C_3_N_4_ helped distribute the stress under extreme deformation, with the storage moduli 32 times stronger than a conventional radical initiators.

The photocatalytic ability of g‐C_3_N_4_ could also aid in the biomimetic photosynthetic activity of NADH‐dependent enzymatic reactions with triethanolamine (TEOA) as electron donors (Figure 5D).^[^ ^76^ ^]^ The self‐assembled peptide (Fmoc‐diphenylalanine, Fmoc‐FF) was hybridized with g‐C_3_N_4_ to assist the exfoliation of g‐C_3_N_4_ nanosheets, which enhanced the photocurrent density of Fmoc‐FF/g‐C_3_N_4_ hydrogel, due to an increased number and extended lifetime of charge carriers.^[^ ^76^ ^]^ TEOA was oxidized with the help of g‐C_3_N_4_ nanosheets and gave electrons to the production of NADH by reducing NAD^+^. This hydrogel efficiently transferred photoinduced electrons to regenerate the enzymatically active NADH from NAD^+^ for catalytically synthesizing L‐glutamate.^[^ ^76^ ^]^

The photoinduced electrons of g‐C_3_N_4_ have been utilized in an optically neural stimulating platform with the assistance of graphene oxide to retard the recombination of an electron–hole via effective charge transfer across the heterojunction between g‐C_3_N_4_ and graphene (Figure 5E). In this model, PC12 cells showed enhanced neurite outgrowth under visible‐light irradiation (450 nm) in a spatiotemporal‐controlled manner.^[^ ^77^ ^]^ In addition to neural differentiation, the photoactivated stimulation via g‐C_3_N_4_ nanosheets and two‐photon laser could also accelerate bone regeneration.^[^ ^78^ ^]^ The photocurrent near the cell nuclei could activate a transduction signaling that increased cytosolic Ca^2+^ concentration, leading to cellular proliferation and differentiation toward the bone formation.^[^ ^78^ ^]^ Considering its low cost, low toxicity, and high chemical stability, accelerated investigations of g‐C_3_N_4_ in light‐responsive biomaterials can be expected in the future.

### 3.4 Transition metal oxides (TMOs)

**Figure 6:**
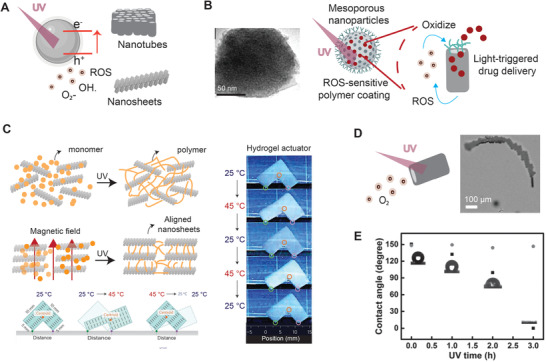
TiO_2_ nanoparticles are used as photodynamic agents and photoinitiators under UV light. A) TiO_2_ nanoparticles have various shapes, including nanosphere, nanotube, nanosheet, and mesoporous nanoparticles. B) SEM image of TiO_2_ mesoporous nanoparticles. TiO_2_ mesoporous nanoparticles can load drugs and be modified with ROS‐sensitive polymers for the use of light‐responsive drug delivery. The production of ROS by UV irradiation can oxidize the ROS‐sensitive polymers to trigger the release of the drugs loaded in the pores of the nanoparticles. Adapted with permission.^[^ ^83^ ^]^ Copyright 2015, Elsevier. C) TiO_2_ nanosheets can trigger radical polymerization to form nanocomposite hydrogels. The nanosheets can be aligned by a magnetic field to form an anisotropic hydrogel, which can be further used as a thermal‐responsive actuator. Adapted with permission.^[^ ^87^, ^88^ ^]^ Copyright 2015, Springer Nature. D) The photocatalytic ability of TiO_2_ nanotubes can generate oxygen bubbles to propel itself to move with the direction guided by light. SEM image of TiO_2_ nanotubes propelled by bubbles under the irradiation of UV light. Adapted with permission.^[^ ^89^ ^]^ Copyright 2015, John Wiley & Sons. E) UV Light can alter the hydrophilicity of TiO_2_‐based nanostructured surfaces. Adapted with permission.^[^ ^90^ ^]^ Copyright 2015, John Wiley & Sons.

TMOs have drawn much attention for photocatalytic applications due to their conductivity, adjustable bandgap, and light absorption of semiconducting characteristics. During photocatalysis, photonic energy was transferred via light illumination with its energy equal or greater than the bandgap energy of TMOs, which can excite electrons to jump from the valence band (VB) to the conduction band (CB), and then generate electronic holes in the VB. Subsequently, a subset of the photogenerated electrons and holes migrates to the surface and proceeds with various redox reactions with the adsorbed molecules on the TMOs surface to produce radicals, such as superoxide ion (O_2_^•−^), or hydrogen peroxide (H_2_O_2,_ hydroxyl radicals OH^•^) (**Figure** **6**A). TMOs have a broad structure variety because of their ability to form phases of varying oxygen and metal ratios resulting in the different bandgap, specific light wavelength absorption, and photocatalytic efficiency.^[^ ^7^, ^79^ ^]^ For instance, titanium dioxide (TiO_2_) is a typical n‐type semiconductor material which has four polymorphs, for which only anatase and rutile are efficient enough for practical photocatalytic applications due to the broader bandgap.^[^ ^80^ ^]^ TiO_2_ is the most popular TMOs, especially in biomedical research for applications of surface coating, anticancer agents, and substrates for stem cell expansion, because of its low cost, excellent biocompatibility, and chemical stability.^[^ ^81^ ^]^

TiO_2_ has been considered a promising material for PDT.^[^ ^82^ ^]^ For example, highly crystalline TiO_2_ was synthesized by flame spray pyrolysis with a predominant anatase phase (87%) and a small content of rutile (13%), which had high UV responsiveness.^[^ ^82^ ^]^ Also, the abundant hydroxyl groups on the surface of TiO_2_ nanoparticles could be used to conjugate with amino acids. The amino acids were used for the agglomeration of TiO_2_ nanoparticles and the surface functionalization of fluorescein isothiocyanate dyes and drugs.^[^ ^82^ ^]^ After a short period of UV irradiation (20 s), the amino acid L‐lysine was decomposed and oxidized into pipecolinic acid, a cyclic compound of lysine, leading to the photocleavage of the agglomerated nanoparticles and the release of drugs from the TiO_2_ surface.^[^ ^82^ ^]^ TiO_2_ has various structures; for instance, mesoporous TiO_2_ nanoparticles allow anticancer drugs to be loaded into the nanometer‐sized pores (2–3 nm).^[^ ^83^ ^]^ Polyethyleneimine (PEI) was coated on the mesoporous TiO_2_ nanoparticles as a gate to block the diffusion of drugs. After exposure to UV, the PEI layer could be oxidized and destroyed by free radicals, thus triggering drug release on demand (Figure 6B).^[^ ^83^ ^]^ Also, doping TiO_2_ nanoparticles in wound dressing hydrogel have been shown to have efficient antibacterial effects and lead to accelerated wound healing.^[^ ^84^ ^]^ However, their wide optical band gaps (3.2–3.2 eV) restrict their applications in clinical areas, since UV has a limited penetration depth through the skin. Moreover, owing to that UV is required to activate TiO_2_, the long‐term and indoor use of TiO_2_ is compromised. Hence, novel nanocomposites and different types of TiO_2_ have been developed to reduce the bandgap, allowing TiO_2_ to be activated from visible to NIR light. Hydrogenated black TiO_2_ nanoparticles with a narrow bandgap in ≈2.32 eV and enhanced NIR light absorption were also explored as PDT and PTT agents for cancer therapy.^[^ ^85^ ^]^

Titania nanosheets can also be used as photoinitiators for radical polymerization due to their strong photocatalytic ability (Figure 6C).^[^ ^86^ ^]^ Moreover, titania nanosheets also acted as a physical crosslinker by noncovalently absorbing polymers, which gave the nanocomposite hydrogel good mechanical strength.^[^ ^86^ ^]^ In addition, titania nanosheets were fixed at the crosslinked points in a 3D polymer network, allowing micropatterning of hydrogels with an excellent spatial resolution. Interestingly, titania nanosheets with high aspect ratios could be co‐facially oriented by electrostatic repulsion in a hydrogel actuator that could thermally control its movement by altering the distance between the nanosheets (Figure 6C).^[^ ^87^ ^]^ Photolatently modulable hydrogels have shown various responsiveness to photo, temperature, and magnetic field, which might have great potential for 4D cell culture and bioprinting.^[^ ^87^, ^88^ ^]^

Moreover, most of TMOs are polymorphism, which can be flexibly synthesized in different morphologies, such as nanofibers, nanorods, nanobelts, and nanowires. TiO_2_ can also be processed into a tubular structure as photocatalysis‐propelled micro/nanomotors. In a 1% H_2_O_2_ aqueous environment, TiO_2_ microtubes can generate O_2_ bubbles that were ejected from the TiO_2_ microtube to push itself forward by the photocatalytic decomposition of H_2_O_2_ (Figure 6D).^[^ ^89^ ^]^ This tubular design has great potential for the combination therapy of PDT and drug delivery light‐guided nanovehicles.^[^ ^89^ ^]^

The surface wettability is also a hot field of TiO_2_ research. The wettability of TiO_2_ film could be reversibly controlled between superhydrophilic and superhydrophobic conditions under alteration of UV light irradiation and long‐term dark storage, independent of their photocatalytic activities (Figure 6E).^[^ ^80^, ^90^ ^]^ There has been much research devoted to a better understanding of the mechanism of light‐responsive wettability change. Atomic rearrangement of the TiO_2_ surface is one of the well‐accepted theories because the phase transformation of TiO_2_ from 101 to 001 was observed after the UV treatment.^[^ ^90^ ^]^ However, the photocatalysis of TiO_2_ can also simultaneously induce the switching wettability via the degradation of low energy hydrocarbon groups and the formation of high energy hydroxyl groups on the TiO_2_ surface. Potential biomedical applications have been investigated, such as self‐cleaning coating, surface patterning, microfluidic devices, and cell behavior modulation.

A multifunctional platform was established using the extreme wettability contrast on the TiO_2_ nanotube array surface.^[^ ^91^ ^]^ Self‐assembling monolayers of silanes were used to mask TiO_2_ film before the UV irradiation to perform the photocatalytic cleavage of the TiO_2_‐silane bond.^[^ ^91^ ^]^ Then, the wettability pattern on the TiO_2_ nanotube array surface could be treated as a template to selectively deposit various materials and biomolecules, based on the nature of the absorbed materials on the TiO_2_ film. Moreover, calcium phosphate nanocrystals, growth factors, and cancer drugs could be immobilized on a specific location, which makes it a powerful tool for high‐sensitivity cell assays, which are used for drug screening and bio‐chips. For example, cells prefer to attach and proliferate on a hydrophilic region. TiO_2_ film with extreme contrast of wettability could also be used as a template for fabricating nanoscaled multiphasic particles. Self‐assembled polymers were dip‐coated on the wettable domains defined by the silane mask geometry, thus enabling the fabrication of complex shapes and sizes as small as 25 nm.^[^ ^92^ ^]^ Multiphasic assemblies could also be built in a bottom‐up manner by depositing polymers on top of each other, which provided the particles with multifunctional capabilities as drug, fluorescence, and functional nanoparticle carriers.

Although TiO_2_ film has the unique characteristic of wettability change upon UV irradiation, not much research is focused on its biomedical applications due to the slow recovery rate from super hydrophilicity to high hydrophobicity. The crystalline phases of TiO_2_ not only significantly affect the photocatalytic activity but also the photoinduced hydrophilicity. In contrast to the anatase phase, the wettability of rutile nanodot films almost showed no change after UV illumination. However, the rutile nanodot films still could induce greater cell detachment than anatase, caused by the damage of the proteins’ secondary structure due to different electron transfer properties. Thus, understanding the mechanism of light‐responsive surface properties can help us to better utilize TiO_2_ film for biomedical applications.

TMOs are receiving significant attention in recent years for their potential applications in the ablation of cancer cells. The band structure positions and bandgap energies of TMOs are crucial for their uses. For instance, ZnO, TiO_2_, SnO_2_, Fe_2_O_3,_ and ZrO_2_ cannot initiate photopolymerization by visible light for potential applications of tissue engineering and bioprinting. The bandgaps of SnO_2_, ZnO, and TiO_2_ are larger than 3.2 eV, giving them a thermodynamically favorable photocatalysis performance, but the restricted range of light absorption and high electron–hole pair recombination rates of these TMOs still hinder their photocatalytic performances. For example, the vanadium (V)‐doped SnO_2_ nanoparticles were more active as a catalyst than pure SnO_2_ because the estimated band gaps were reduced from 3.65 to 2.60 eV.^[^ ^93^ ^]^ Although Fe_2_O_3_ (2.2 eV) and V_2_O_5_ (2.8 eV) have a low bandgap and are responsive to visible light, their relatively low CB position cannot effectively consume photoinduced electrons, resulting in a low production of radicals.^[^ ^79^, ^94^ ^]^ Therefore, most of the biomedical applications of these TMOs focused on antibacterial activities and ex vivo environments. For instance, ZnO, TiO_2_, MoO_3,_ and WO_3_ have already been shown to have good photoinduced antibacterial activity.^[^ ^95^, ^96^ ^]^ In recent years, due to the improvement of nanomaterial synthesis, the hybrid structures, surface modifications, and defect control of TMOs have been developed in various fields, such as water splitting and pollute degradation. Though their potential as in vivo therapy agents is not clear yet, research has provided substantial references for expanding their applications into the field of PTT and PDT. WO_3_ nanoparticles were modified by the dopamine‐conjugated hyaluronic acid to be more biocompatible and improve tumor targetability.^[^ ^97^ ^]^

Photocatalytic efficiency is also affected by electron localization or the numbers of active sites on the surface, which can be tuned by stoichiometry, elemental doping, environmental stimuli, surface area, and film thickness.^[^ ^98^, ^99^ ^]^ It was found that the WO_3_ gel exhibited a sustainable release of H_2_O_2_ induced by localized electrons due to the rapid surface temperature increase by the sunlight‐induced photothermal conversion.^[^ ^99^ ^]^ Also, doping metal ions to TMOs could generate oxygen vacancies to maintain the charge balance, which could result in the improvement of the photocatalytic performance. For example, doping metal ions on the surface of WO*_x_* could trap and localize electrons around the dopants and enhance the photoinduced electron density on the active sites.^[^ ^100^ ^]^ The active sites have a more robust electron‐giving ability indicating more intense charge transfer with reactants and ROS generation. The improved photocatalytic efficiency implies the expected use of TMOs for PDT in the future.

Moreover, several novel hypoxic metallic oxides, including WO_3−_ *_x_*, MoO_3−_ *_x_*, and RuO_2_, were investigated as novel PTT materials.^[^ ^96^, ^101^, ^102^ ^]^ Hydrous RuO_2_ nanoparticles were synthesized by a facile hydrothermal treatment and surface‐modified with a polyvinylpyrrolidone (PVP) coating, which showed a good dispersion in saline solution and high PTT performance.^[^ ^103^ ^]^ The stoichiometric ratios between metal atoms and oxygens can also enhance LSPR for PTT. Oxygen‐deficient nanocrystals, such as TiO_2−_ *_x_*, WO_3−_ *_x_*_,_ and MoO_3−_ *_x_*, are typical nonstoichiometric TMOs found to have active LSPR in the NIR region.^[^ ^102^, ^104^ ^]^ Nonstoichiometric TMOs possess mixed valence ions that cause an intervalence charge‐transfer transition, leading to the shift of the absorption peaks from visible to NIR region and the unique character of the outer‐d valence electrons. Nonstoichiometric TMOs have higher charge carrier densities than other identified TMOs, which can also increase the intensity of the NIR plasmonic peaks.

The development of these plasmonic nonstoichiometric TMOs has opened new possibilities for TMOs to be utilized in cancer therapy. MoO_3–_ *_x_* was synthesized and modified by polyethylene glycol (PEG) via a facile one‐pot template‐free hydrothermal route, which was demonstrated to be valuable for drug delivery, contrast imaging, and PTT because of its high NIR absorption.^[^ ^102^ ^]^ The reduction ability of PEG played a crucial role in the mediation of oxidation states of Mo ions leading to a strong LSPR effect.^[^ ^105^ ^]^ Therefore, the tunable and strong NIR absorption makes nonstoichiometric TMOs good candidates for novel biomedical strategies, including NIR imaging and PA‐guided chemo‐photothermal therapy.

The development of NIR‐responsive photothermal TMOs is still at an early stage. Compared to AuBNs and CBNs, TMOs hold the advantage of low cost and facile synthesis. However, information about their toxicity and immune response is still limited. Besides anticancer and antibacterial abilities, TMOs also might have potential applications for regenerative medicine. The degradation products of TMOs contain rare elements that can play a crucial role in the human body and can be combined with optical cell modulation treatment to develop novel phototherapy for tissue regeneration. Understanding their mechanisms of photodynamic and photothermal effects, assessing the impact of structure, crystalline phase, and stoichiometry on the optical properties of TMOs, and investigating their toxicity and stability in physiological environments are future directions in order to develop biomedical applications of TMOs.

### 3.5 Transition metal dichalcogenides (TMDs)

TMDs are composed of a transition metal atom from groups 4 to 7 (Mo, W, Ta, Re, and Mn) and two chalcogenides (S, Se, Te). Although oxygen (O), sulfur (S), selenium (Se), and tellurium (Te) all belong to the chalcogen group, TMDs feature very different properties and stoichiometries compared to TMOs. Due to the improvement of the exfoliation technique and surface functionalization of 2D materials, more and more TMDs nanosheets have been discovered, which exhibit massive differences in optical, mechanical, and electronic properties from their bulk materials. Exfoliated TMDs have attracted increasing attention for electronic, photonic, and catalysis applications.^[^ ^8^, ^106^ ^]^ Because of the quantum confinement effect, the electronic band structures of TMDs shift from indirect to direct, and their bandgap energies increase concurrently along with the reduction of the TMDs’ thickness. The bandgap energies of TMDs are generally within the range of 1.6–2.4 eV, which are smaller than that of TMOs, and thus suitable for visible light catalysis.^[^ ^79^ ^]^ However, unlike TMOs that can be used as photocatalysis agents alone, TMDs are generally combined with other photocatalysts as co‐catalysts and electron sinks for retarding photogenerated electron–hole recombination, which are widely used for PDT and biosensing. For biomedical applications, TMD nanosheets that contain elements with a high atomic number are frequently used as contrast agents for computed tomography (CT) imaging. Moreover, many TMD nanosheets are active NIR‐responsive materials, which could potentially be used for PTT and PA imaging. Therefore, TMD nanosheets have become one of the most popular 2D nanomaterials after the discovery of graphene in biomedical research.

**Figure 7:**
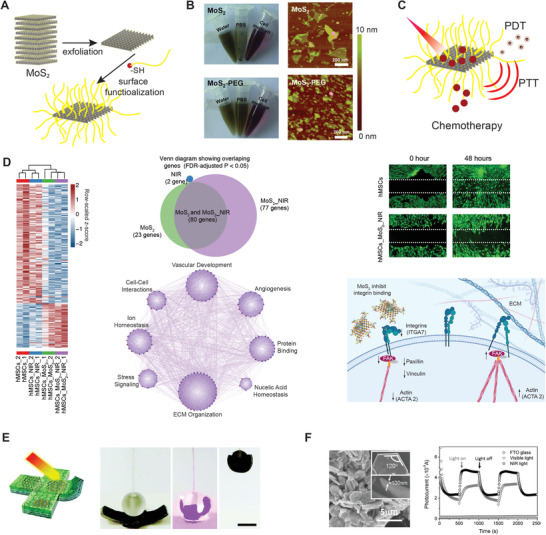
Various types of 2D TMDs are used to develop light‐responsive biomaterials, especially for cancer therapy and soft actuators. A) Bulk MoS_2_ are exfoliated into nanosheets and further functionalized by thiol‐terminal polymers to increase their aqueous stability.^[^ ^112^ ^]^ B) Optical images of MoS_2_ and polyethylene glycol‐modified MoS_2_ (PEG‐MoS_2_) dispersed in water, phosphate‐buffered saline (PBS), and cell medium. AFM images of MoS_2_ and PEG‐MoS_2_. Adapted with permission.^[^ ^112^ ^]^ Copyright 2014, John Wiley & Sons. C) MoS_2_ nanosheets have been used for photodynamic, photothermal, and chemotherapy, which are frequently used for synergistic cancer therapy. D) Cellular effect of NIR‐stimulated MoS_2_ on gene expression determined using RNA‐seq. Photomodulation using MoS_2_ nanosheets suppresses cellular migration by inhibiting integrin.^[^ ^117^ ^]^ Copyright 2020, National Academy of Sciences (NAS) of the United States of America. E) The photothermal effect and 2D structure of TMDs nanosheets allow the fabrication of light‐responsive and anisotropic hydrogel actuators. Adapted with permission.^[^ ^120^ ^]^ Copyright 2016, John Wiley & Sons. F) WS_2_ nanosheets with a unique bandgap are found to be active photocatalysts under visible and NIR irradiation. Adapted with permission.^[^ ^125^ ^]^ Copyright 2014, John Wiley & Sons.

Among all the TMDs materials, molybdenum disulfide (MoS_2_) has been intensively investigated for biomedical applications due to the negligible cytotoxicity and superior NIR absorption.^[^ ^107^, ^108^ ^]^ As a member of graphene‐analog materials, MoS_2_ inherits some common features from graphene, with the ultrahigh surface‐to‐volume ratios and the nature of the hydrophobic surface allowing MoS_2_ to absorb biomolecules, hydrophobic drugs, and genes for drug delivery. In addition, MoS_2_ has a higher NIR absorption than GO and reduced GO, leading to better PTT performance.^[^ ^109^ ^]^ Thus, MoS_2_ can combine PTT and chemotherapy by introducing efficient exfoliation and surface modification. The conventional method of lithium intercalation (Morrison method) was frequently utilized to break the interlayer force of MoS_2_ bulk materials by immersing MoS_2_ in n‐butyllithium solution in hexane and sonicating it under the protection of nitrogen gas (**Figure** **7**A).^[^ ^110^, ^111^, ^112^ ^]^ The assistance of sonication could shorten the process from 2 days to hours, but results in a decrease in the lateral sizes of the nanosheets. The sonication‐assisted solvothermal methods can be done by dispersing MoS_2_ in organic solvents (e.g., *N*‐methyl‐pyrrolidone and oleum oil) with high boiling points in the sonication bath.^[^ ^113^ ^]^ However, due to their high boiling points, it is hard to obliterate the organic solvents, which will cause cytotoxicity. Therefore, more biocompatible exfoliation options have been developed by using bio‐friendly agents such as bovine serum albumin (BSA), and a mixed solvent of water and ethanol, despite the decreased concentration of nanosheets.^[^ ^114^, ^115^ ^]^ The nonpolar parts of BSA, attached to MoS_2_, and the polar part of BSA, interacted with water, act as a natural surfactant.^[^ ^114^ ^]^ However, these methods can only stabilize the nanosheets in water temporarily, due to the dense mass of nanosheets. Thus, after the exfoliation and cleaning processes, surface modification a very crucial step to improve the physiological stability, biocompatibility, and performance of TMD nanosheets for therapeutic applications.

The interaction between MoS_2_ and sulfur can be used for the conjugation of molecules containing thiol or disulfide terminal groups at the atomic defects of nanosheets. The mechanism and binding strength of anchoring thiolated molecules on MoS_2_ are still not clear, but it has already become a general way to functionalize MoS_2_. Lipoic acid‐modified PEG, containing a disulfide group on its end‐group, can attach at the defect sites of MoS_2_, preventing the aggregation and improving the aqueous stability of MoS_2_ (Figure 7A,B)_._^[^ ^112^ ^]^ Moreover, folic acid, PEI, and hyaluronic acid were grafted with thiol‐terminated groups to functionalize MoS_2_ for promoting cellular uptake of anticancer nanocarriers, gene delivery agents, and miRNA detection nanoprobes (Figure 7C).^[^ ^110^, ^112^, ^113^, ^116^ ^]^ Once obtaining physiological stability, MoS_2_ could be further loaded with anticancer drugs, photodynamic agents, or genetic materials to perform versatile biomedical applications. Also, molecules (e.g., DNA oligonucleotides, PEG) conjugated on MoS_2_ via vacancy‐driven modification can be released in a glutathione reducing environment.

For chemo‐photothermal cancer therapy, cancer drugs can be attached to the MoS_2_ surface via *π*–*π* staking and hydrophobic interaction, and the drugs could be released on‐demand by PTT using continuous NIR to inhibit the cancer drug resistance. While under pulsed NIR, PA tomography can be performed with significant signal enhancement compared to traditional PA agents, due to the extremely high light‐to‐heat conversion efficiency.^[^ ^113^ ^]^ Therefore, the doses of MoS_2_ used in the current study for PTT are much lower than those of nano‐graphene and reduced GO. When the surface‐functionalized MoS_2_ nanosheets were delivered to the intracellular endosome, PTT could trigger the endosomal escape and release of nanosheets. In contrast, the intracellular reducing agent in the cytoplasm, glutathione, could degrade the disulfide bond between MoS_2_ and thiolated molecules, resulting in the enhancement of MoS_2_, drugs, and gene accumulated in the cells.

Apart from cancer therapeutics, the photothermal effect of MoS_2_ has shown strong potential in regenerative medicine with the ability to modulate cellular activity.^[^ ^117^ ^]^ The high surface area of MoS_2_ nanosheets facilitates protein adsorption and cellular adhesion, leading to the localization of MoS_2_ nanosheets on the cell surface. The role of light‐actuation of MoS_2_ on stem cells was investigated via whole transcriptome sequencing (RNA‐seq) to provide global and unbiased snapshot of gene expression (Figure 7D).^[^ ^117^ ^]^ MoS_2_ nanosheets and subsequent stimulation with NIR light is shown to influence ≈103 and 157 genes in human mesenchymal stem cells (hMSCs), respectively. The results showed that most of the differentially regulated genes are related to cellular migration and wound healing. A significant reduction in integrin signaling was observed due to MoS_2_ and NIR treatment. The combination of MoS_2_ and NIR light may provide new approach to control and regulate cellular functions for regenerative medicine as well as cancer therapy.

MoS_2_ is also reported to have the ability of PDT. MoS_2_ nanoflowers with high NIR absorption and peroxidase‐like activity could decompose a low concentration of H_2_O_2_ to generate hydroxyl radicals.^[^ ^118^ ^]^ Moreover, it has also been reported that MoS_2_ QDs could produce ^1^O_2_ under 630 nm laser light, implying that MoS_2_ might have the potential of allowing combinational therapy of PDT and PTT without adding other photosensitizers.^[^ ^119^ ^]^ Since MoS_2_ is an active PTT agent, it has also been developed as NIR‐responsive actuators and hydrogel microvalves.^[^ ^109^, ^120^ ^]^

The successful investigation of MoS_2_ has demonstrated the potential of MoS_2_‐analog TMDs for light‐responsive therapy. WS_2_ and MoSe_2_ also have superior photo‐to‐heat conversion efficiency, which has been applied to PTT and smart actuators.^[^ ^121^, ^122^ ^]^ In recent research, more biocompatible, facile, and functional methods of exfoliation were developed for biomedical applications of TMDs. MoS_2_ was simultaneously exfoliated and noncovalently factionalized via the imidazole rings of polymeric ionic liquid, which could be further crosslinked by a one‐step quaternized reaction to form an NIR‐responsive smart hydrogel (Figure 7E).^[^ ^120^ ^]^ Besides, the amphiphilic nature of PVP assists the exfoliation of MoSe_2_, which endowed the nanosheets with good hydrophilicity and stability in aqueous solution.^[^ ^122^ ^]^ Among TMD materials, MoSe_2_ nanosheets have a distinct absorption peak at ≈808 nm, yet MoS_2_ has only an NIR absorbing band, indicating the enormous potential of MoSe_2_ to be an efficient PTT agent. Moreover, MoSe_2_ nanoflowers were proven to be a potential material for PDT, with the ability of NIR‐mediated photocatalysis.^[^ ^124^ ^]^ The bandgap of MoSe_2_ nanoflowers is about 1.24 eV, which is smaller than MoS_2_, allowing deep‐tissue NIR light therapy and the stimulation of electron transition for the production of •OH radicals under NIR light.

Tungsten (W) dichalcogenides are also a popular group of nanomaterials for phototherapy with high photothermal and photodynamic responsiveness to NIR light. However, the popularity of Mo dichalcogenides overshadowed that of W dichalcogenides due to their heavier atomic weight and limitedly industrial availability. The applications and characteristics of W and Mo disulfide are similar, but the exfoliation and functionalization methods of MoS_2_ might not be efficiently applied to WS_2_. For example, the functionalization of WS_2_ through thiol‐terminated molecules has not been reported yet. Despite these disadvantages, WS_2_ and other rarely used TMDs have their unique properties that deserve further investigation.

For instance, WS_2_ nanosheets are the first TMDs nanosheets reported to have the ability of photocatalysis from UV to NIR without adding other photocatalysts and causing photothermal effects (Figure 7F).^[^ ^125^ ^]^ The photo‐oxidation efficiency of MoS_2_ is low due to a small indirect bandgap (1.17 eV) and a low valance band maximum (*E*_VB_(vs NHE) = 1.4 eV). In contrast, the narrow bandgap (1.35 eV) allows WS_2_ to expand the light absorption region to 910 nm. Also, the higher valance band maximum (*E*_VB_(vs NHE) = 1.71 eV) of WS_2_ gives it a sufficient driving force for high NIR light photocatalytic activity, as its narrow bandgap is still within the NIR‐triggerable range. Moreover, the relatively long lifetime of photogenerated charge carriers is also beneficial for the photocatalytic process. Unlike the monolayered or multi‐layered structure of WS_2_, the unique structure of WS_2_ nanosheets with 100 nm thickness did not generate heat under NIR irradiation. It suggests that the structure of TMDs might be a critical factor to tune their photoresponsive behavior.

Therefore, in the group of TMDs, there are still many narrow bandgap semiconductors that could be considered for NIR photocatalysis with a suitable band structure. Controlling the structures or elemental doping might be a good start to investigate the possibility of using TMDs as NIR‐responsive PDT agents. The NIR photocatalysis will be more biocompatible and practical for applications of PDT and light‐guided drug delivery, without the need for heating and UV irradiation. Moreover, MoS_2_ and WS_2_ both have low cytotoxicity and genotoxicity.^[^ ^126^ ^]^ The functionalized TMDs can be further used in 4D cell culture and cell behavior modulation via photocurrent, mild heat, and redox stimulation. In summary, research into TMDs is still at the initial stage due to their variety in element combinations, surface functionalization, processing techniques, and structure design. Overall, TMDs have the potential to become a leading topic in the field of light‐responsive biomaterials.

### 3.6 Mono‐Elemental Nanosheets

**Figure 8:**
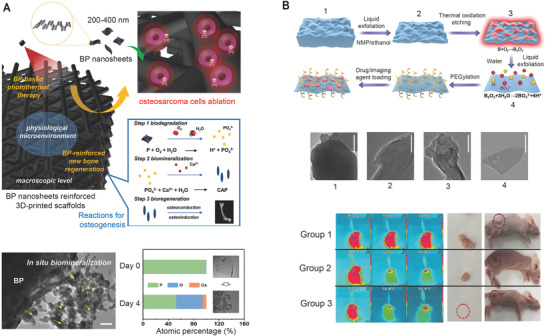
Mono‐elemental nanosheets are emerging light‐responsive nanomaterials. Black phosphorous (BP) and boron nanosheet (BNS) are frequently used mono‐elemental nanosheets due to the biocompatibility of their degradation products and superior photothermal effects. A) BP nanosheets‐reinforced 3D‐printed scaffolds used for treatment of osteosarcoma. The BP nanosheets enable photothermal ablation of osteosarcoma. Subsequently, the degradation of BP nanosheets results in the release of PO_4_^3−^ that promotes in situ biomineralization. Adapted with permission.^[^ ^130^ ^]^ Copyright 2018, John Wiley & Sons. B) Schematic illustration and SEM images of the stepwise preparation of 2D PEG‐modified BNS. (1: boron sheets after dispersed in NMP and ethanol solution; 2: liquid exfoliated BNS in NMP and ethanol solution; 3: thermal oxidation‐etched BNS; 4: liquid exfoliated BNS in water.) Infrared thermographic maps show the time‐dependent changes of temperature in the tumor‐bearing mice after three different treatments. Digital photos of tumors excised from representative mice show the potent therapeutic effects of the synergistic photothermal‐chemotherapy from the BNSs. (Group 1: BNS + doxorubicin; Group 2: BNS + NIR; Group 3: BNS + doxorubicin + NIR.) Adapted with permission.^[^ ^131^ ^]^ Copyright 2018, John Wiley & Sons.

Black phosphorus (BP) nanosheet is a newly researched 2D and metal‐free nanomaterial used for PTT, PDT, bioimaging, and drug delivery (**Figure** **8**A). BP has a layer‐dependent direct bandgap varying from 0.3 to 2.0 eV, allowing it to have a broad light absorption across the UV and NIR regions. BP nanosheets also have a high NIR extinction coefficient and photothermal conversion efficiency. Most importantly, since phosphorous plays a crucial role in many physiology activities and makeup ≈1% of the total body weight as a bone constituent, BP can be easily degraded as nontoxic phosphate and phosphonate in the physiological environment. Therefore, BP has been deemed as a harmless and degradable biomaterial for light‐responsive applications.

Like for other 2D TMD nanosheets, liquid phase ultrasonication‐assisted exfoliation is one of the most popular methods to prepare BP nanosheets. Bulk BP powder is mixed with the ice bath of organic solvents (e.g., isopropanol and *N*‐methyl‐2‐pyrrolidone) and exfoliated by a probe sonication process. The negatively charged surface of BP nanosheets could be further functionalized with positively charged ligands and polymers to increase their biocompatibility and stability in an aqueous environment. BP QDs loaded in poly(lactic‐co‐glycolic acid) (PLGA) nanospheres could be used for photothermal cancer therapy.^[^ ^127^ ^]^ BP will rapidly be oxidized and degraded in water in the presence of oxygen. Hence, the PLGA nanospheres could prevent BP QDs from degradation to control the release rate of BP's degradation products without causing apparent toxicity and inflammation.

The high photothermal conversion efficiency (38.8%) and large surface‐to‐volume ratio of BP nanosheets were utilized to build a novel light‐controlled drug‐delivery system.^[^ ^128^ ^]^ The BP nanosheets converted NIR light into heat that softened and melted a drug‐loaded agarose hydrogel to increase the permeability of drug diffusing into surrounding cancer cells. Besides BP, phosphorus has two other primary allotropes, which are red and white phosphorus. Red phosphorus, which is more cost‐effective than BP, has been used as an efficient photothermal coating for in vivo antibiofilm applications.^[^ ^129^ ^]^ Moreover, a BP‐loaded 3D‐printed scaffold for bone regeneration not only enabled the photothermal ablation of osteosarcoma but also accelerated the recovery of bone defects (Figure 8A).^[^ ^130^ ^]^ The BP nanosheets were oxidized and degraded into phosphate ions that could induce calcium‐extracted biomineralization and promote osteogenesis. Unlike TMDs, which contain heavy metal atoms, BP nanosheets with excellent biocompatibility and biodegradability are suitable for developing cancer therapies and hard tissue scaffolds.

Recently, boron nanosheets (BNSs) were introduced as a novel mono‐elemental, nonmetal nanomaterial used for multimodal‐imaging‐guided cancer therapy (Figure 8B).^[^ ^131^ ^]^ Elemental boron has similar chemical and physical properties to carbon, while boron nanomaterials may theoretically have superior chemical stability, thermoelectricity, and conductivity. Boron is also a trace mineral in our body and plays a crucial role in bone growth, wound healing, and anti‐inflammatory processes.^[^ ^132^ ^]^ However, due to the rich bonding configurations among B atoms, bulk boron materials cannot be exfoliated by the traditional methods used to exfoliate graphene and TMDs, which are naturally layered structures. Therefore, the techniques of thermal oxidation and liquid exfoliation were combined to obtain BNS (Figure 8B).^[^ ^131^ ^]^ In this process, BNSs were first functionalized by amine‐PEG and loaded with doxorubicin and imaging molecules. Moreover, the high photothermal conversion efficiency (42.5%) allowed it to be used as a great photothermal and PA imaging agent. The in vivo results showed the synergistic chemo‐photothermal therapy significantly inhibited the tumor growth.

Boron and phosphorous are both essential elements for supporting cell metabolism. Through investigating the optical properties of these elemental nanomaterials, BP and BNS can be further developed as novel light‐responsive nanomaterials for tissue engineering. Moreover, due to the improvement of nanosheet exfoliation techniques, more and more 2D layered elemental materials and minerals, such as antimonene and biotite nanosheets, were explored for their applications in photonic nanomedicine.^[^ ^133^ ^]^ However, the limited options for noncovalent surface modifications for these materials may compromise its further applications. Therefore, to promote the light‐responsive biomedical applications of mono‐elemental nanosheets, the development of novel methods to functionalize the nanosheets covalently is necessary.

### 3.7 Upcoversion nanoparticles (UCNPs)

**Figure 9:**
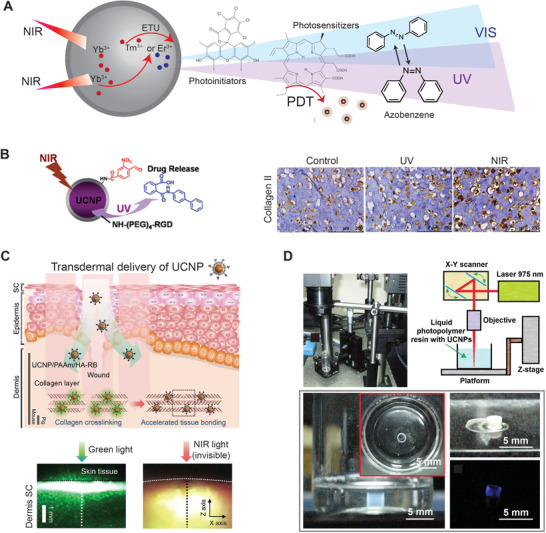
UCNPs are a group of nanomaterials enabling conversion of NIR light to UV or visible light. A) The UV or visible light emission of UCNPs can trigger photoinitiators, photosensitizers, azobenzene molecules, or any other UV and visible light‐sensitive molecules for various light‐responsive applications. B) NIR‐triggered release of small molecules directs stem cell differentiation by the kartogenin‐conjugated UCNPs. Immunohistochemical staining of hydrogel sections against type II collagen shows that NIR light could trigger the release of kartogenin from the UCNPs and induce significant chondrogenic differentiation of hMSCs with high Collagen II expression in vivo. Adapted with permission.^[^ ^144^ ^]^ Copyright 2016, Elsevier. C) Transdermal delivery of UCNPs and photosensitizers, RB, produce collagen radicals beneath the skin under NIR light for accelerating tissue bonding. Optical images show the propagation of green light and light converted by the UCNPs upon invisible NIR light illumination. Adapted with permission.^[^ ^145^ ^]^ Copyright 2017, American Chemical Society. D) High‐resolution 3D photopolymerization assisted by UCNPs circumvents the limited working distance of UV photopolymerization for large‐scale printing. Adapted under the terms of the Creative Commons Attribution 4.0 International License.^[^ ^146^ ^]^ Copyright 2018, Springer Nature.

UCNPs are a unique group of nano‐transducers that have a fascinating ability to convert long‐wavelength light into short‐wavelength light for a wide range of phototherapy applications.^[^ ^134^ ^]^ In the last decade, UCNPs are of great use in the biomedical field due to the photonic conversion of NIR to visible or UV light, which accelerated the development of in vivo photonic medicine by overcoming the limited penetration depth of UV and visible light. Energy transfer up‐conversion is the dominant process of up‐conversion efficiency, which includes the absorption of the same energy photon by each of the two neighboring ions and a nonradiative energy transfer process (**Figure** **9**A).^[^ ^135^ ^]^ One of the ions is promoted to a higher energy level which results in the emission of higher energy photons, while the other ion relaxes back to the ground state. Therefore, UCNPs have three components that resemble two different kinds of lanthanide dopant ions embedded in a host lattice. Fluoride‐based host materials are one of the most common types of UCNPs due to the relatively low photon energies, thus providing long lifetimes of excited electrons states. In the dopant ions, the one that donates energy is called sensitizer, and the other that emits visible or UV light is called activator. The concentration of the sensitizer would be higher than the activator to decrease cross‐relaxation energy loss. Based on these criteria, NaYF_4_ doped with Yb^3+^ as the sensitizer, and Er^3+^ or Tm^3+^ as the activator is the most common compositions of UCNPs.^[^ ^136^ ^]^ Many factors can improve the up‐conversion luminescence efficiency of UCNPs, including particle diameter, crystal host materials, crystal phase, lanthanide ion dopants, surface coating, and dispersion medium, which have been extensively discussed in other review papers.^[^ ^134^, ^137^ ^]^

UCPN is a therapeutic tool for deep tissue therapy and brain stimulation. Optogenetic therapy is one of the most promising strategies for treating neurological disorders, but now it requires the insertion of invasive optical fibers to superficial brain structures due to the short penetrating‐depth of blue light, which limits its use in the clinic. In contrast, UCNP‐mediated optogenetics could be feasible for transcranial stimulation of deep brain neurons with the high transmittance of NIR light in brain tissue and the improved up‐conversion efficiency of UCNPs. For example, the blue‐emitted core–shell NaYF_4_:Yb/Tm nanocrystals were decorated with silica (NaYF_4_:Yb/Tm@SiO_2_) or poly(acrylic acid) (PAA) (NaYF_4_:Yb/Tm@PAA) to increase long‐term utility and biocompatibility.^[^ ^138^ ^]^ The emitted blue light could activate channelrhodopsin‐2 (ChR2), a commonly used light‐gated ion channel, to modulate dopamine release of dopaminergic neurons. Also, an advantage of UCNPs is tunable wavelengths of emission light by selective lanthanide‐ion doping. Thus, UCNP is a multi‐wavelength light source that can control various neural activities as a multifunctional toolbox, such as activation of inhibitory neurons in the medial spectrum, silenced seizure by inhibition of hippocampal excitatory cells, and recall fear memory by triggering granule cells in the hippocampus.^[^ ^138^ ^]^

UV and blue light‐emitted UCNPs have provided a promising solution for developing PDT for deep tumors since direct NIR‐responsive PDT reagents are rare. A dual‐stimuli‐responsive nanocomposite of a pH‐responsive polymeric ligand, a photosensitive chlorin e6 (Ce6), and an NIR‐responsive UCNP could prevent the inevitable side effects in surrounding healthy tissues of tumors.^[^ ^139^ ^]^ When the nanocomposites were transported to the acidic tumor microenvironment (pH ∼ 6.5), the imidazole groups of the pH‐responsive polymeric ligand would quickly reverse the surface charge to positive, which disassemble the aggregated polymeric ligand and dequench the photoactivity of Ce6. Therefore, only the nanocomposite in the NIR exposed region and the acid microenvironment of the tumor simultaneously can be triggered, which enhanced the selectivity of PDT.^[^ ^139^ ^]^ Also, for the PDT of viral infections, UCNPs could be further modified to increase target specificity to virus‐infected cells by bioconjugation of antibody specific for infection‐induced proteins.^[^ ^140^ ^]^ By carrying the photosensitizer, UCNPs could photodynamically treat virus‐infected tissue without causing collateral damage to other healthy cells, as a promising strategy of antiviral phototherapy.^[^ ^140^ ^]^ Due to the NIR‐responsive ability, UCNPs can also be used as on‐demand nanocarriers with the assistance of mesoporous‐silica coating. The mesoporous‐silica shell can be easily coated on UCNPs through typical silica sol–gel reactions, which provide the space for drug and biomolecule loading.^[^ ^141^ ^]^ The repeated stirring motion of azobenzene molecules triggered by the emitted blue and UV light of UCPNs propelled the drugs out from the pores of mesoporous silica. As for tumor immunotherapy, PDT cannot only kill cancer cells but can also promote an antitumor immune response. The released tumor antigens from the mesoporous‐silica coating of UCNPs synergistically enhanced immunotherapy efficacy, combined with the ROS generated by photosensitizers merocyanine 540, under NIR 980 nm irradiation.^[^ ^142^ ^]^ Similarly, UV cleavable linkers such as *o*‐nitrobenzyl moieties and succinimidyl groups, crosslinked in the hydrogel networks contained UCNPs, can perform photodegradation by NIR light.^[^ ^143^ ^]^ The incorporation of an UV cleavable linker selectively quenched the UV emission bands of UCNPs, and the up‐converted NIR emission allowed for deep tissue imaging.

Unlike a thermosensitive and pH‐sensitive hydrogel, the photodegradable hydrogel allowed the encapsulation and delivery of macromolecules and proteins without compromising their bioactivities. Although UV light can be harmful to biomolecules, compared to stimuli like heating or ROS, the low intensified and localized UV light emission, which is also partially absorbed by the photocleavable linkers, makes it more suitable to be applied in tissue engineering. hMSCs play a crucial role in regenerative medicine, which usually needs inducing agents to control differentiation. As a classic example of dynamic materials for regenerative medicine, UCNPs conjugated with the peptide (Cys‐Arg‐Gly‐Asp, CRGD) and the differentiation‐inducing protein kartogenin via a nitrobenzoic acid photocaged linker can be taken up by hMSCs (Figure 9B).^[^ ^144^ ^]^ The on‐demand NIR‐triggered release of kartogenin could induce chondrogenic differentiation of the subcutaneously implanted hMSCs, enhancing neocartilage formation in vivo. The immunohistological analysis showed that NIR‐treated hydrogels which contained kartogenin‐conjugated UCNPs exhibited significantly more intense type II collagen compared with the two control groups. This research showed the great potential of utilizing UCNPs in time‐dependent 4D cell culture and bioprinting by localized up‐converted UV and visible light emission.

Photochemical tissue bonding is a tissue repair technique that uses a dye‐assisted photochemical technique to crosslink proteins on a tissue surface for wound closure. Rose Bengal (RB) dye can be activated by green light at 540 nm to take electrons from the collagen and generate collagen radicals where the covalent crosslinks formed between collagens for the tissue bonding. However, the short penetration depth of green light limited its use in deep tissue areas. Therefore, UCNPs can replace the function of the optical fibers or waveguides to emit the light to dyes for healing the tissue inside the wound (Figure 9C).^[^ ^145^ ^]^ Moreover, due to the relatively small size of UCNPs compared to fibers, UCNPs conjugated with RB and hyaluronic acid could be noninvasively delivered into deep tissue. In the optical images, NIR light was converted to visible light under the skin, while green light was blocked. (Figure 9C) The wound healing performance of this noninvasive tissue repair technique was even better than conventional suture, as confirmed by in vivo animal tissue bonding tests and ex vivo tensile strength tests.

NIR light‐induced radical polymerization could also be a revolutionary approach for 3D bioprinting, combining the benefits of the high resolution of laser scanning photolithography and the good biocompatibility of NIR light. Recently, a high‐resolution 3D photopolymerization was developed in which UCNPs, dispersed in a liquid mixture of photocurable composition, emitted UV light to activate the photoinitiators under moderate intensities of NIR excitation below 10 W cm^−2^ for free‐radical polymerization (Figure 9D).^[^ ^146^ ^]^ This research found that the heterogenous distribution of photoluminescence intensity around UCNP would cause different morphologies of polymer growth from the surface of a single UCNP due to the different polymer growth kinetics. Moreover, it required a minimum concentration of UCNPs to grow geometrically connected polymeric objects for 3D printing. Therefore, to apply this concept to the photolithography bioprinting for complex tissue structures or bio‐microfluidic devices, there are still several problems to overcome. The UV light conversion efficiency of UCNPs is around 2% at an NIR intensity of 25 W cm^−2^_,_ which is still far from the safe range of laser intensity usage in biomedical applications, especially at 980 nm.^[^ ^146^ ^]^ The biocompatibility and photophysical complexity of UCNPs have not been fully investigated. Overall, the unique up‐conversion ability of UCNPs will ensure the growth of its research efforts in the field of light‐responsive biomaterials.

### 3.8 Other Types of Light‐Responsive Materials

Semiconducting nanoparticles, or QDs, have unique optical and electronic properties that differ from larger particles, which have been widely investigated in the field of bioimaging.^[^ ^119^, ^147^ ^]^ For phototherapy applications, NIR‐responsive QDs have attracted tremendous interest due to the therapeutic window. It has been reported that cadmium (Cd)‐containing QDs can kill a wide range of multidrug‐resistance bacterial clinical isolates by altering the cellular redox state.^[^ ^148^ ^]^ Moreover, the complexity of the intracellular redox environment indicates the possibility of stimulating other cellular responses besides cell toxicity. Therefore, the Cd‐containing QDs can also generate specific light‐activated redox species to control the proliferation of bacteria by tuning the redox potential of the QDs through size‐dependent quantum confinement.^[^ ^148^ ^]^

The feasibility of tuning the bandgap of QDs shows their great potential use in the study of the effect of redox states of on living systems. More recently, novel types of QDs were emerged in biomedical research to avoid the toxicity problems of Cd‐contained QDs by replacing Cd by other benign elements. Graphene QDs and carbon QDs are particularly attractive with the typical optical properties and photocatalytic functions of conventional QDs, but with improved biocompatibility and photostability. The graphene QDs have also shown great antibacterial properties through ROS‐mediated photodynamic effects.^[^ ^149^ ^]^ The graphene QDs can also process photothermal and photodynamic actions simultaneously and can be tracked with laser irradiation for post‐therapy imaging.^[^ ^150^ ^]^ There are numerous types of QDs synthesized by downsizing the nanoparticles of CBNs, TMOs, TMDs, g‐C_3_N_4_, and mono‐elemental nanosheets, which indicates their great potential in biomedical research.^[^ ^119^, ^151^ ^]^

Silicon nanoparticles have also been reported to possess photothermal and photodynamic capabilities under NIR light due to the quantum confinement effect.^[^ ^152^ ^]^ Porous silicon nanoparticles can function as photosensitizers to generate singlet oxygen against cancer cells.^[^ ^153^ ^]^ Another report showed that the silicon nanoparticles could convert absorbed NIR light into localized heat for PTT.^[^ ^154^ ^]^ The silicon nanoparticles are biodegradable and biocompatible, with a degradation product of nontoxic orthosilicic acid, which can be excreted from a body via urine.^[^ ^155^ ^]^ The porous silicon nanoparticles can also construct a hydrogel network by generating singlet oxygen to initiate the polymerization of poly(ethylene glycol) double acrylates by NIR light.^[^ ^156^ ^]^

Some multinary metal chalcogenide nanocrystals recently draw much attention for PTT because of their strong LSPR absorbance in the NIR region. For instance, the relatively small bandgap (0.16 eV) of CuFeSe_2_ provides higher light‐to‐heat conversion efficiency compared to that of other multinary nanocrystals with a wide bandgap.^[^ ^157^ ^]^ CuFeSe_2_ was recently used to develop an oxygen‐independent free‐radical‐generated nanosystem by incorporation of 2,2‐azobis[2‐(2‐imidazolin‐2‐yl)propane]‐dihydrochloride (AIPH).^[^ ^157^ ^]^ The rapid growth of cancer cells in tumor tissue could form a hypoxia microenvironment which is extremely resistant to oxygen‐dependent PDT since the ROS production of PDT is primarily dependent on the level of oxygen concentration.^[^ ^158^ ^]^ AIPH is an azo compound that can produce alkyl free radical under the stimulation of heat. Therefore, by carrying AIPH, CuFeSe_2_ could use photothermal activity to trigger the disintegration of AIPH to produce free radicals, as synergistic phototherapy for treating hypoxic tumors.^[^ ^157^ ^]^

## 4 Emerging Approaches and Future Prospects

A range of light‐responsive inorganic nanomaterials are discussed for various biomedical applications. These nanomaterials have multiple advantages and limitations that are listed in **Table** **1**. The development of light‐responsive inorganic nanomaterials can eliminate the limitations of current phototherapy because of their long‐wavelength light responsiveness and superior stability in the physiological environment. The PDT agents accumulated in tumors could aid in processing several treatments until the patient recovered to minimize the injection pain. However, the organic photocatalysts now used in the clinical setting still suffer from photobleaching, which decreases its efficacy of PDT gradually. Instead, the inorganic photocatalysts not only has better aqueous stability and less photobleaching effect but also can be facilely functionalized to perform multiple applications other than PDT. The surface modifications of natural polymers or biomolecules on the PDT agents allowed them to target cancer cells and accumulate more in tumors. For instance, MoS_2_ is one of the most popular TMDs used in the biomedical field because of the convenience of a surface modification via thiol–MoS_2_ interactions to form Mo—S covalent bonds. Combining with the ROS sensitive molecules and polymers, the PDT agents can generate the radicals by light to alter the surface properties or trigger drug release on‐demand.^[^ ^159^, ^160^ ^]^ Also, the polymeric networks containing ROS‐responsive cleavable units can integrate with the PDT agents to form photodegradable hydrogels for drug and cell delivery.

**Table 1:** Overview of inorganic light‐responsive nanomaterials with representative materials, applications, advantages, and disadvantages

Material categories	Representative materials	Applications	Advantages	Limitations
CBNs	Graphene, graphene oxide, carbon nanospheres, CNTs, nanodiamonds, carbon QDs	PTT, antibacterial materials, neuron/cardiac cell stimulation, light‐responsive drug delivery, actuator, dynamic biomaterial	Chemical inertness, biocompatibility, large surface area to absorb light and molecules	Poor solubility in the physiological environment, limited photodynamic activity, limited size, and shape control
AuBNs	Gold nanorod, nanoparticle, nanochinus, nanocross, nanostar, nanocage (various shapes of AuBNs based on the synthesis methods)	PTT, antibacterial materials, neuron/cardiac cell stimulation, light‐responsive drug delivery, light‐guided nanovehicle, actuator, dynamic biomaterial	Chemical inertness facile functionalization, biocompatibility, tunable sizes, and absorption peak	Less drug‐loading capacity, limited photodynamic activity
g‐C_3_N_4_	Carbon nitride nanosheet, nanotube, mesoporous carbon nitride	Antibacterial materials, neuron/bone stimulation, photocatalyst	Chemical inertness, high hardness, low toxicity, ease of preparation	Not responsive to long‐wavelength light
TMOs	ZnO, TiO_2_, black TiO_2_, SnO_2_, Fe_2_O_3,_ ZrO_2_, V_2_O_5_, MoO_3_, WO_3_, WO_3−_ *_x_*, MoO_3−_ *_x_*, RuO_2_	PTT, PDT, antibacterial materials, actuator, light‐guided nanovehicle, light‐responsive drug delivery, dynamic biomaterial, photocatalyst	Adjustable bandgap, efficient photoactivity, large surface area, low cost	Uncertain toxicity of degradation products, weak photoactivity to long‐wavelength light, limited surface functionalization strategies
TMDs	MoS_2_, WS_2_, MoSe_2_	PTT, PDT, antibacterial materials, actuator, light‐responsive drug delivery	High heat conversion efficiency, high‐specific surface area, abundant catalytically active sites	Poor solubility in the physiological environment, hazardous synthetic methods, limited surface functionalization strategies
Mono‐elemental nanosheets	Black phosphorus (BP), boron nanosheet (BNS)	PTT, light‐responsive drug delivery	Inherent degradability, high heat conversion efficiency, large surface area, biocompatibility	Weak photodynamic activity, limited surface functionalization strategies
UCNPs	NaYF_4_:Yb/Tm, NaYF_4_:Yb/Tm (various types of UCNPs based on the lanthanide ion doping)	Optogenetic therapy, light‐responsive drug delivery, dynamic material, photochemical tissue bonding	High up‐conversion efficiency, high resistance to photobleaching, narrow emission bandwidth	Uncertain toxicity of degradation products, limited functionalization strategies

With conjugation of other stimuli‐responsive materials, inorganic phototherapy agents would be able to target cancer cells in both external stimulation and microenvironment responsive fashion. For example, a high concentration of reductive glutathione and hypoxic microenvironment in tumors could compromise the anticancer efficiency of PDT. Thus, oxidizing nanomaterials such as MnO_2_ nanosheets could integrate with PDT agents to react with glutathione and reduce the consumption of ^1^O_2_.^[^ ^161^ ^]^ As more characteristics of different types of cancer are discovered, more sophisticated and personalized designs of phototherapy nanoplatforms could be developed. The synergistic phototherapy composed of PDT, PTT, chemotherapy, gene delivery, or immunomodulating would be a future strategy to develop treatments for highly specific types of cancer, which would rely heavily on the interdisciplinary collaboration between medical and material researchers.^[^ ^108^, ^162^ ^]^

Recently, heterojunction nano‐semiconductors are being widely investigated in developing novel photocatalysts to produce solar fuel cells with higher energy conversion rates.^[^ ^163^ ^]^ The heterojunction structure could spatially separate the photogenerated electron–hole pairs and prevent them from fast recombination, which leads to higher photocurrent and photocatalytic activity. Moreover, the heterojunction structures can also alter the bandgap and further widen the absorbance range from UV light, which only accounts for 2–4% of solar energy to visible or even NIR light. In the case of TiO_2_ and MoS_2_ heterostructure, the MoS_2_ nanosheets act as an efficient light harvester, and the heterostructure interface realizes charge separation by suppressing the electron–hole recombination.^[^ ^164^ ^]^ In addition, various carbon nanomaterials have been integrated with TiO_2_ to enhance photocatalysis. SWNTs/TiO_2_ nanocomposites displayed higher visible photooxidation and photoreduction activities, compared to graphene/TiO_2_ nanocomposites due to its hybrid metallic and semiconducting nature.^[^ ^165^ ^]^ The charge transfer interaction on the interface of metallic and semiconductor nanomaterials is the main factor for tuning photocatalytic response. Moreover, the connection between photosensitizers will also affect their performance. For example, noncovalent and covalent bonding have different charge transferring rates, which leads to different photocatalytic activities. Therefore, these concepts are also applicable to the next‐generation photoresponsive nanomaterials used in the PDT, PTT, and nonoptical genetic modulation.

UCNPs provide a great platform to use emerging light‐responsive nanomaterials with exciting features confined by the limited penetration depth of UV light in clinical use. The effective PDT and PTT agents require high absorption cross‐sections and low scattering losses in the NIR region, but most of the available photosensitizers can only absorb UV and visible light. Thus, the NIR‐responsive UCNPs could trigger the photodynamic activity of TiO_2_ or other UV‐responsive metal oxides via NIR‐to‐UV up‐conversion for deep‐tissue PDT.^[^ ^166^ ^]^ For instance, UCNPs coupled with ZnFe_2_O_4_, a semiconductor catalyst, have shown enhanced photodynamic activity through introducing of the Fenton reaction.^[^ ^167^ ^]^ The coated mesoporous ZnFe_2_O_4_ shell with a redox pair (Fe^2+^/Fe^3+^) could produce more virulent ˙OH after absorbing UV light emitted by the UCNPs.^[^ ^167^ ^]^ Apart from the PDT, the photothermal effect of nanomaterials could also be optimized by introducing heterostructures. The coupling effect of a gold nanorod and copper sulfide could enhance the local field and optical absorption at the surface, and decrease the scattering loss of the gold nanorod core, to approach higher photothermal conversion efficiency.^[^ ^168^ ^]^ Through rationally designing the heterostructures of photoresponsive nanomaterials, not only the efficiency of light energy utilization can be improved, but also the multifunctional photoresponsive nanomaterials can be further developed. These inorganic nanomaterials can simultaneously be also considered as crosslinkers in hydrogels, carriers in drug delivery devices, and bioactive agents in tissue engineering, which the small molecules are unable to do.

The photoresponsive chemistry has great contributions in developing 4D cell culture through dynamically controlling the degradation rate of a hydrogel or patterning the functional proteins at a specific spatial‐temporal region. The complex multi‐vascular networks of organs were demonstrated recently by using synthetic food dyes as biocompatible photoabsorbers to enable stereolithographic production of photopolymerized hydrogels.^[^ ^169^ ^]^ Moreover, proteins can also be patterned in the hydrogels to stimulate cell growth and migration via the orthogonal light chemistry. However, so far, all these reported chemical mechanisms are only responsive to UV and visible lights, which may cause damage to cells in the long term and have limited curing depth for increasing the size of 3D‐printed tissue. Therefore, developing a red light or NIR light‐responsive 4D cell culture hydrogel is a solution to overcome these limitations and approach the size of human organs. Therefore, UCNPs will be still playing an essential role in these fields due to its long‐term photostability and NIR‐to‐UV conversion ability. Thus, besides increasing the efficacy of the energy conversion of UCNPs, the long term and system biocompatibility of UCNPs should also be investigated.

The technique of 4D cell culture could also be applied to bioprinting and can be deemed as a type of photoresponsive 4D bioprinting, wherein the biophysical and biochemical microenvironment could be tuned by light after printing. Another type of photoresponsive 4D printing is to use light to trigger the deformation or movement of the hydrogel scaffolds after printing. This technique allows the 4D bioprinting to print more complex structures close to native tissues, such as liver and heart. Temperature is the most commonly used stimulus to deform printed scaffolds via using the thermal‐responsive polymer. Then, light can precisely control the deformation spatially and temporally by the incorporation of photothermal nanomaterials in the scaffolds. For the 4D bioprinting, based on the maturation of engineered tissue constructs, the ROS generated by photosensitizers could also be used to oxidize and cleave the ROS‐linkers to perform the localized drug release and alter the structure of the hydrogel networks to guide cell behaviors.^[^ ^159^ ^]^ It can be expected that the use of light‐responsive inorganic nanomaterials will be an emerging trend in 4D bioprinting research.

## 5 Conclusion

The inorganic PTT and PDT agents are useful in the fight against drug‐resistance cancers and to ease patient's pain by precision therapy. More NIR‐responsive PTT and PDT agents were developed in recent years, with the goal of improving ROS and heat conversion efficacy. This research pushed phototherapy one step further toward the treatment of deep tissue cancers. Moreover, multifunctional PTT and PDT agents cannot only ablate cancer cells but also accelerate tissue recovery. Photocurrent and mild photothermal stimulation are found to activate signaling pathways in bone marrow stem cells, neuron cells, and cardiac cells to assist tissue regeneration. In regenerative medicine, the role of inorganic nanomaterials is more likely to be considered as energy transducers to transform photonic energy to heat, radicals, or different wavelengths of light to trigger other stimuli‐responsive materials to process “treatments.” Light‐guided nanovehicles propelled by gas, concentration, and heat gradients are expected to deliver drugs to a specific location via light‐guided control and imaging. Moreover, light‐responsive materials provide a precise, efficient, and feasible platform to control the biophysical and biochemical properties of dynamic biomaterials. The spatiotemporal tuning and patterning of the cellular microenvironment are essential to mimic the complex structure of native tissue. Additionally, the development of 4D cell culture and bioprinting could be done through the precise control of photoinducible, biorthogonal chemistry. Although light‐responsive inorganic nanomaterials are still rarely reported in 4D cell culture, the ability to transform photonic energy with superior stability and efficacy makes them critical components in further regenerative research of dynamic biomaterials. Overall, from PDT, PTT, and light‐guided devices to optical cell modulation and 4D cell culture, the drastic increase in research into light‐responsive inorganic nanomaterial shows their promising future.

## Conflict of Interest

The authors declare no conflict of interest.
